# Peripheral monocyte–derived cells counter amyloid plaque pathogenesis in a mouse model of Alzheimer’s disease

**DOI:** 10.1172/JCI152565

**Published:** 2022-06-01

**Authors:** Ping Yan, Ki-Wook Kim, Qingli Xiao, Xiucui Ma, Leah R. Czerniewski, Haiyan Liu, David R. Rawnsley, Yan Yan, Gwendalyn J. Randolph, Slava Epelman, Jin-Moo Lee, Abhinav Diwan

**Affiliations:** 1 Department of Neurology,; 2 Hope Center for Neurological Disorders, and; 3 Department of Pathology and Immunology, Washington University School of Medicine, St. Louis, Missouri, USA.; 4 Department of Pharmacology and Regenerative Medicine, University of Illinois College of Medicine, Chicago, Illinois, USA.; 5 Department of Medicine, Washington University School of Medicine, St. Louis, Missouri, USA.; 6 John Cochran VA Medical Center, St. Louis, Missouri, USA.; 7 Department of Surgery, Division of Public Health Sciences, Washington University School of Medicine, St. Louis, Missouri, USA.; 8 Peter Munk Cardiac Centre, University Health Network, Toronto, Ontario, Canada.

**Keywords:** Neuroscience, Alzheimer disease, Monocytes

## Abstract

Microglia, the parenchymal tissue macrophages in the brain, surround amyloid plaques in brains of individuals with Alzheimer’s disease (AD) but are ineffective at clearing amyloid to mitigate disease progression. Recent studies in mice indicate that microglia are derived exclusively from primitive yolk sac hematopoiesis and self-renew without contribution from ontogenically distinct monocytes/macrophages of definitive adult hematopoietic origin. Using a genetic fate-mapping approach to label cells of definitive hematopoietic origin throughout life span, we discovered that circulating monocytes contribute 6% of plaque-associated macrophages in aged AD mice. Moreover, peripheral monocytes contributed to a higher fraction of macrophages in the choroid plexus, meninges, and perivascular spaces of aged AD mice versus WT control mice, indicating enrichment at potential sites for entry into the brain parenchyma. Splenectomy, which markedly reduced circulating Ly6C^hi^ monocytes, also reduced abundance of plaque-associated macrophages of definitive hematopoietic origin, resulting in increased amyloid plaque load. Together, these results indicate that peripherally derived monocytes invade the brain parenchyma, targeting amyloid plaques to reduce plaque load.

## Graphical abstract

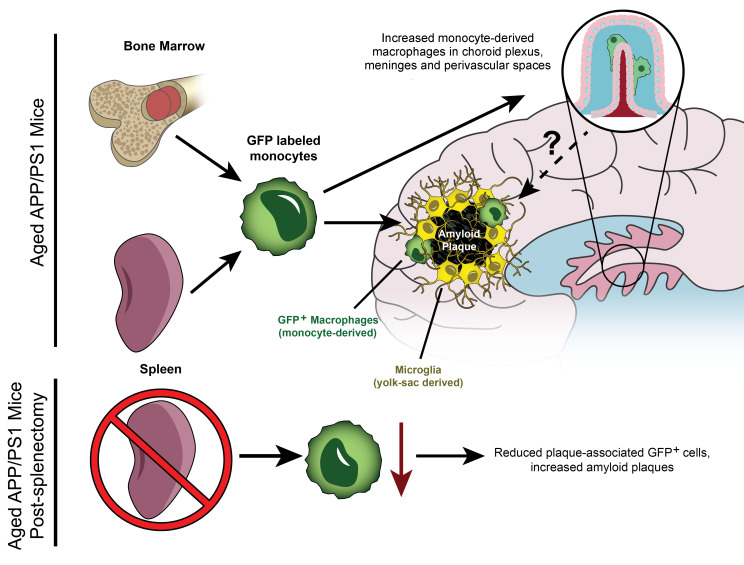

## Introduction

In Alzheimer’s disease (AD), impaired clearance of amyloid β (Aβ) peptide results in its extracellular accumulation and concentration-dependent aggregation into amyloid plaques with neuronal toxicity (1). Microglia, the parenchymal tissue macrophages in the brain, are derived from yolk sac myeloid precursors via primitive hematopoiesis (2) and self-renew without contribution from circulating monocytes (3, 4). Microglia degrade Aβ and phagocytose amyloid material but have been variously characterized as dysfunctional owing to amyloid engorgement (5), dispensable for progression of plaque pathology (6), or as having a limited role in enveloping amyloid plaques to limit local Aβ diffusion and neurotoxicity with advanced AD (7, 8). Consequently, there has been tremendous interest in understanding whether ontogenically distinct peripheral monocytes, i.e., cells derived from definitive hematopoiesis, cross the blood-brain barrier to reinforce microglial function.

Earlier studies have indicated that peripheral monocytes trafficked into the AD brain parenchyma (9, 10); but these observations were confounded by blood-brain barrier disruption induced by irradiation or chemotherapeutic regimens (9). These experimental interventions were determined to be independently sufficient to allow entry of peripheral myeloid cells into the brain (11), regardless of plaque pathology or age (9, 12). Findings of studies implicating recruitment of peripheral monocytes based on cellular markers (13, 14) were subsequently noted to be confounded based on gene expression changes observed in these markers in disease-associated microglia in AD (15–18). Recent attempts to avoid these experimental pitfalls through a parabiosis approach did not demonstrate recruitment of peripheral monocytes; however, these studies were limited to a short 4-week observation window (19). Contemporary fate-mapping approaches have used tamoxifen-induced differential pulse labeling of cells of adult hematopoietic origin (via the CCR2 promoter; ref. 20) in the 5XFAD mouse model, which is characterized by rapid plaque growth (21). CCR2 promoter–driven reporter expression was observed in blood and splenic Ly6c^hi^ monocytes, but not in the brain parenchyma, suggesting that peripheral monocytes did not contribute to brain microglial populations in this model. However, given the short half-life of circulating monocytes (24–48 hours; refs. 22, 23), this intermittent pulse-labeling strategy is expected to be transient, with rapid disappearance of labeled circulating monocytes upon cessation of tamoxifen treatment, resulting in reduced sensitivity for detection of gradual monocyte invasion of the brain compared with a continuous labeling strategy. Alternate approaches to addressing recruitment of peripheral monocyte–derived cells, such as the CXCR4erT2Cre-based fate-mapping strategy in the setting of stroke (24), are not suitable owing to expression of CXCR4 in microglia with amyloid pathogenesis (15, 16, 25). Toward this end, we utilized a fate-mapping approach that constitutively labels monocytes via the *Flt3* promoter (26) throughout the life span of the animal. We chose the APP/PS1 mouse model of AD because it recapitulates the clinical characteristics of slow plaque development (27), which is more reflective of human AD pathogenesis and may provide a broader time window to allow monocyte entry.

## Results

We generated male APP/PS1^mTmG;Flt3-Cre^ and littermate WT^mTmG;Flt3-Cre^ mice with Flt3-Cre–driven expression of a dual-fluorescence mTmG reporter. *Flt3* is solely expressed in all cells of definitive hematopoietic lineage (in fetal liver, adult bone marrow, and spleen) and not in yolk sac–derived microglia (3). Circulating monocytes (Ly6G^lo^CD11b^+^CD115^+^) demonstrated robust Cre-mediated GFP labeling (Supplemental Figure 1, A and B; supplemental material available online with this article; https://doi.org/10.1172/JCI152565DS1), as predicted by expression of Cre via the *Flt3* promoter. Flow cytometric immunophenotyping of cell surface markers in brain macrophage isolates from mice of more than ≥10 months of age revealed GFP expression in CD45^hi^CD11b^+^ cells (Supplemental Figure 2, A and C) that likely included perivascular, meningeal, and choroid plexus macrophages, all of which are known to be maintained, in part, by contributions from adult definitive hematopoiesis-derived cells (4). GFP expression was also noted in a small fraction of CD45^int^CD11b^+^ cells (Supplemental Figure 2, A and D). Importantly, while we did not detect an increase in GFP^+^ cells among all CD45^+^ cellular population (Supplemental Figure 2, A and B), there was a trend toward increased GFP expression in this CD45^int^CD11b^+^ population (Supplemental Figure 2, A and D) in APP/PS1 mice compared with controls. To ascertain the location and characteristics of GFP^+^ cells, we employed immunohistochemistry, a gold-standard technique for examining the regional distribution of macrophage and microglia populations (4).

**Figure 1:**
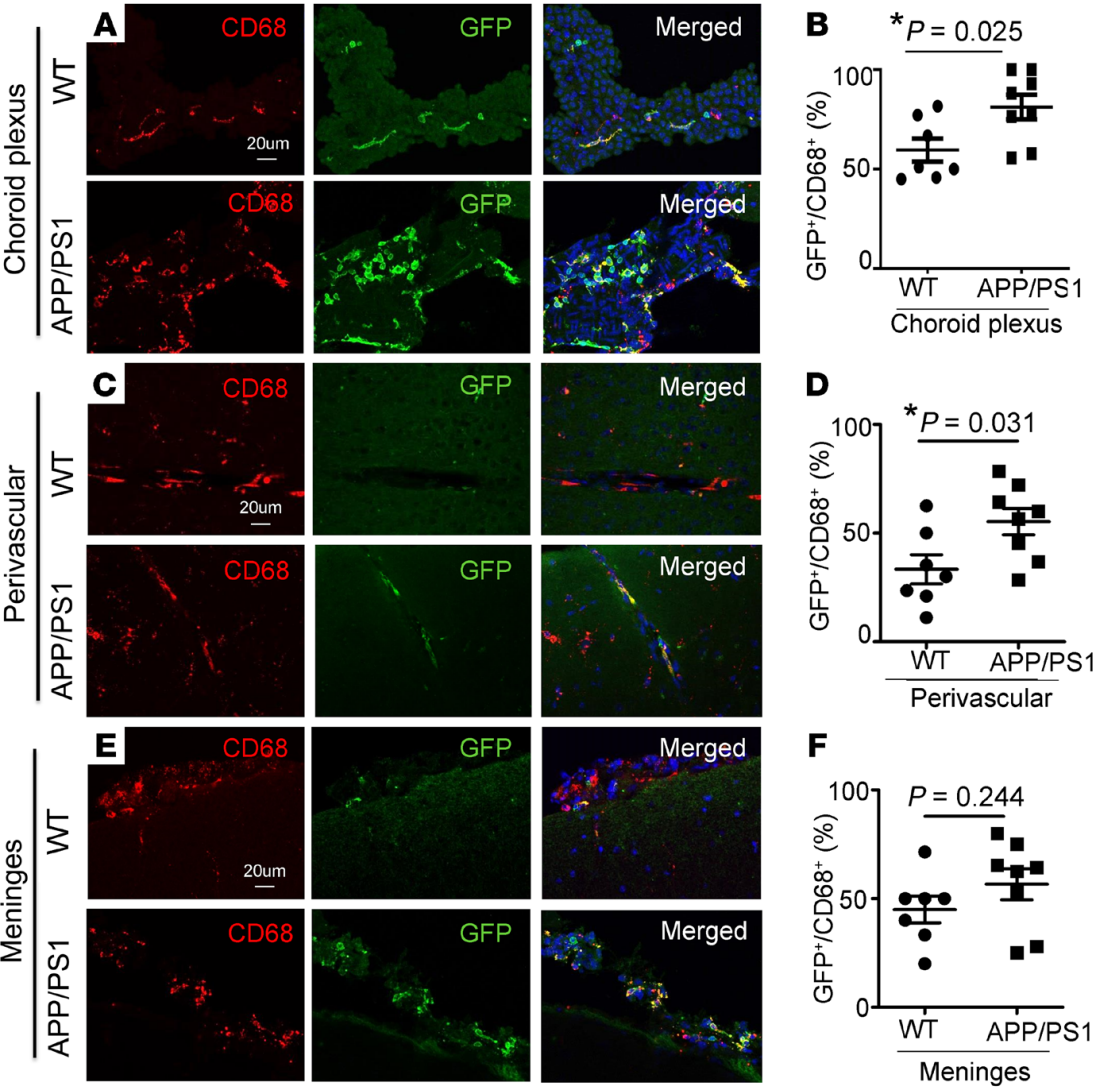
Fate mapping demonstrates an increased contribution of adult hematopoiesis-derived cells to CD68^+^ macrophages in the choroid plexus and perivascular space in APP/PS1 mice. Representative images demonstrating GFP expression in CD68^+^ cells in WT^mTmG;Flt3-Cre^ and APP/PS1^mTmG;Flt3-Cre^ mice, between 10 and 17 months of age, in the (**A**) choroid plexus, (**C**) perivascular space, and (**E**) meninges, (**B**, **D**, and **F**) with quantitation of GFP expression in this cell population in the respective populations. Scale bar: 20 μm (**A**, **C**, and **E**). *n =* 7–8/group. *P < 0.05, *t* test.

**Figure 2:**
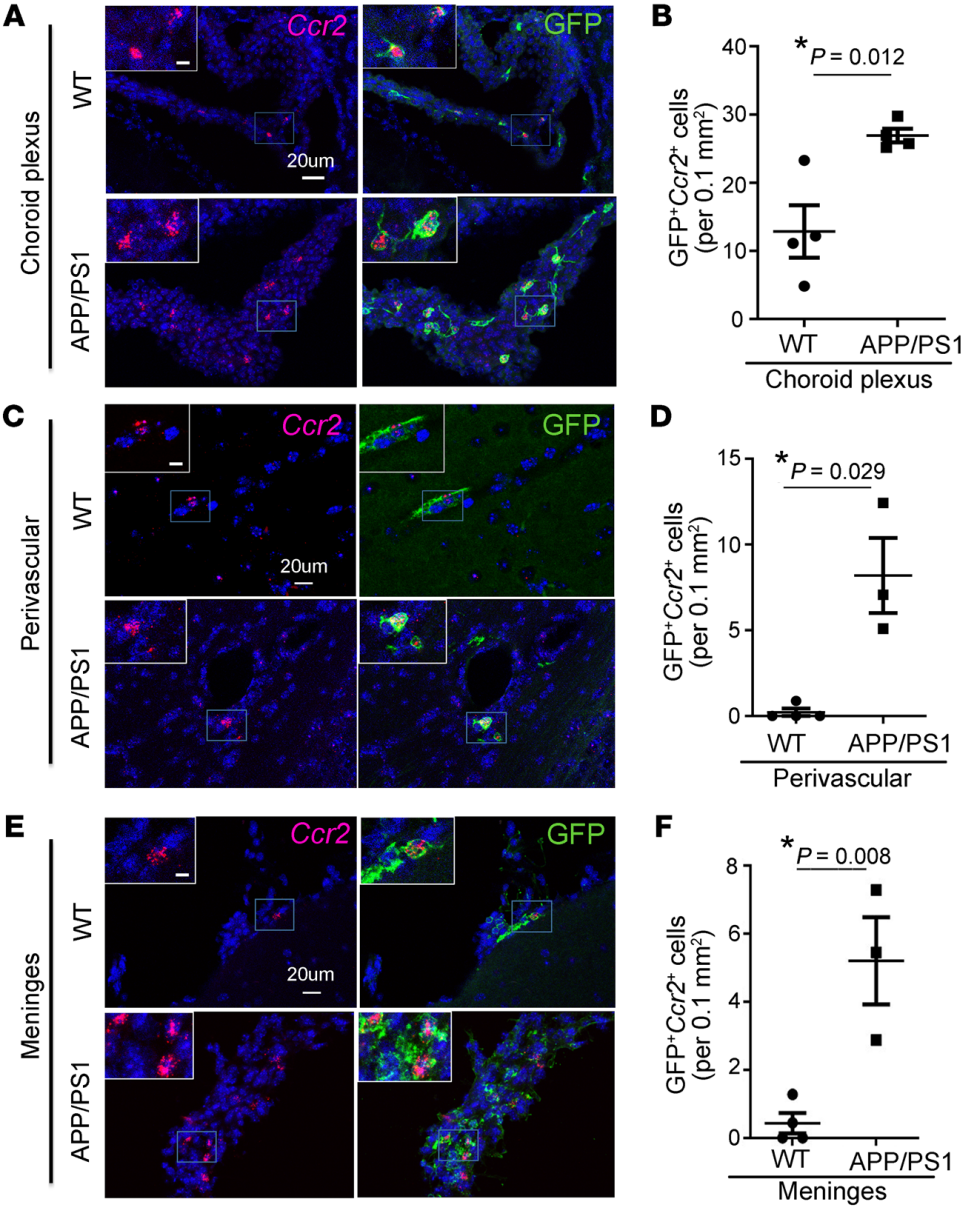
Peripheral monocyte–derived cells are increased in the choroid plexus, perivascular space, and meninges of APP/PS1 mice. Representative images demonstrating GFP^+^*Ccr2*^+^ cells in WT^mTmG;Flt3-Cre^ and APP/PS1^mTmG;Flt3-Cre^ mice, between 10 and 17 months of age, in the (**A**) choroid plexus, (**C**) perivascular space, and (**E**) meninges, (**B**, **D**, and **F**) with quantitation of GFP^+^*Ccr2*^+^ cells in the respective locations. Boxed regions in **A**, **C**, and **E** are shown at higher magnification in insets. Scale bar: 10 μm (inset); 20 μm. *n =* 3–4/group. *P < 0.05, (**B** and **F**) *t* test and (**D**) permutation testing (see Methods).

Histologic examination of brains from aged APP/PS1^mTmG;Flt3-Cre^ and WT^mTmG;Flt3-Cre^ mice (≥10 months of age) revealed GFP-labeled CD45^+^ hematopoietic cells in the choroid plexus, perivascular spaces, and meninges in WT mice (Supplemental Figure 3, A and B, and Supplemental Figure 4, A–F). We found a significant increase in GFP-labeled CD45^+^ (S4A-F) and GFP-labeled CD68^+^ cells (Figure 1) in the choroid plexus (choroid plexus macrophages) and perivascular spaces (perivascular macrophages) in APP/PS1 mice as compared with WT controls. Notably, GFP^+^ cells expressing CCR2 were significantly increased in APP/PS1 mice in the choroid plexus, perivascular spaces, and meninges (meningeal macrophages; Figure 2), as compared with WT controls, indicating their peripheral monocyte origin. These data point to an increased appearance of adult hematopoiesis-derived monocytes/macrophages at these known transit points that permit cellular trafficking into the brain parenchyma (28).

**Figure 3:**
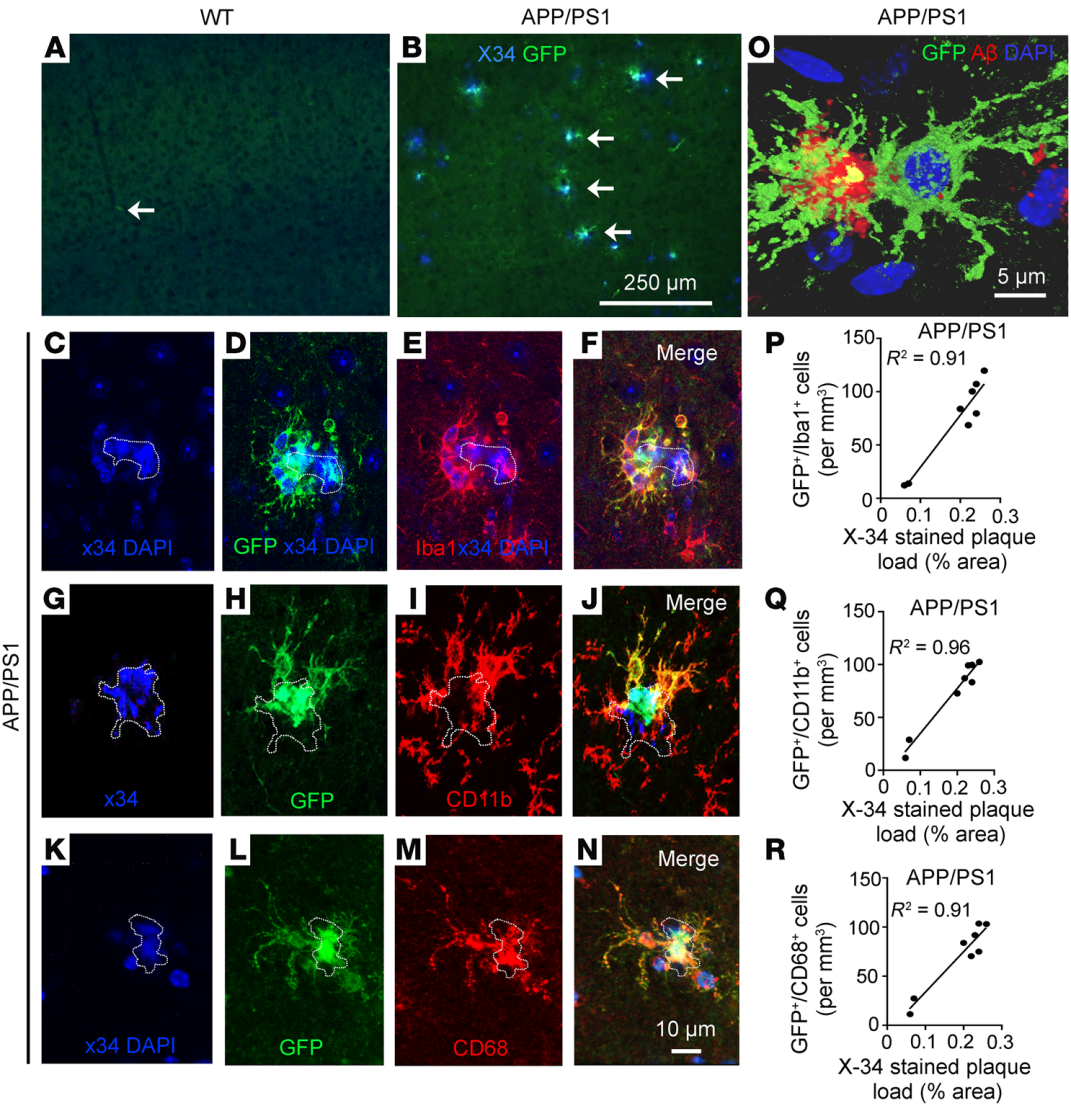
Fate mapping demonstrates peripheral monocyte–derived cells adjacent to amyloid plaques in the brain parenchyma of APP/PS1 mice. (**A** and **B**) Representative cortical sections from aged WT^mTmG;Flt3-Cre^ (*n =* 5) and APP/PS1^mTmG;Flt3-Cre^ mice (*n =* 8) demonstrating that GFP^+^ cells (**A**) are not detected in the WT brain parenchyma and (**B**) are seen adjacent to X-34–stained amyloid plaques in APP/PS1 mice. White arrows point to a GFP^+^ cell in the perivascular space in WT^mTmG;Flt3-Cre^ brain in **A** and to GFP^+^ cells adjacent to X34-labeled amyloid plaques in **B**. Scale bar: 250 μm. (**C**–**N**) Representative cortical sections from aged APP/PS1^mTmG;Flt3-Cre^ mice demonstrating X-34–stained plaque (with DAPI-stained nuclei as shown in **C**, **G**, and **K**; plaque is outlined) (**D**, **H**, and **L**) with GFP expression, (**E** and **F**) which colocalizes with a microglial marker, Iba-1; (**I** and **J**) with CD11b, a marker for activated microglia; and (**M** and **N**) with CD68, a marker for phagocytic cells also present on microglia. Scale bar: 10 μm. Dotted lines outline amyloid plaques. (**O**) A GFP^+^ cell adjacent to amyloid plaque demonstrating colocalization with Aβ (red). Dotted lines outline amyloid plaques. Scale bar: 5 μm. (**P**–**R**) Correlation between the density of GFP^+^ cells expressing (**P**) Iba-1, (**Q**) CD11b, and (**R**) CD68 in the cortex and the amyloid plaque load detected by X-34 staining. Pearson’s coefficient of correlation, R^2^ is shown with *P <* 0.001.

GFP-expressing cells were observed adjacent to amyloid plaques (stained with X-34) in all aged APP/PS1 mice (>10 months of age; Figure 3B and Supplemental Table 1), but they were not detected in WT brain parenchyma (Figure 3A) or in 6-month old APP/PS1 mice (Supplemental Table 1) at a stage prior to development of significant plaque pathology (27). Importantly, GFP-expressing cells were not observed elsewhere in the brain parenchyma of APP/PS1 mice, indicating that these cells home in to the amyloid plaques. GFP^+^ cells expressed Iba1 (Figure 3, C–F), CD11b (Figure 3, G–J), and CD68 (Figure 3, K–N) and demonstrated the lack of expression of astrocyte (GFAP) and neuronal (NeuN) markers (Supplemental Figure 5). These findings indicate that the plaque-associated peripherally derived GFP^+^ monocyte-derived cells had acquired characteristics of macrophages. Because *Flt3*-mediated Cre expression also labels T and B lymphocytes, neutrophils, and NK cells (29), we looked for the presence of these markers in GFP-expressing cells (Supplemental Figures 6–9). While we detected a few GFP^+^ B and T cells in the choroid plexus in both genotypes, consistent with the presence of these cells in normal physiology (18) and the presence of neutrophils and NK cells in the choroid plexus of APP/PS1 mice, plaque-associated GFP^+^ cells did not bear markers for any of these cell types.

We observed that amyloid material colocalized with plaque-associated GFP^+^ cells (Figure 3O), suggesting that monocyte-derived macrophages were intimately interacting with the amyloid plaque. Quantitative examination of APP/PS1 brains revealed that the plaque-associated GFP-expressing cells constitute 5.9% of all CD11b^+^ cells, 4.8% of CD68^+^ cells, and 2.2% of all Iba-1–expressing cells (Table 1 and Supplemental Table 1). There was a strong correlation between plaque load and presence of GFP^+^ periplaque cells (Figure 3, P–R), indicating the extent that plaque pathology may influence the homing signal for these recruited cells. To rule out the possibility that the plaque-associated GFP^+^ peripheral monocytes are observed in APP/PS1 brains because of previously uncharacterized expression of *Flt3* in this milieu, we examined expression of FLT3 protein. We did not find any evidence to support FLT3 expression in WT cortex or its activation in aging mice (Supplemental Figure 10).

Further characterization of plaque-associated GFP^+^ cells demonstrated that these cells coexpressed macrophage and microglial markers such as CD45 and TREM2, a microglial protein implicated in AD pathogenesis (Supplemental Figure 11 and ref. 19). These cells were distinct from the nonplaque-associated GFP^+^ macrophages, as they lacked expression of CD206 (Supplemental Figure 11), a recently described marker enriched in nonparenchymal macrophages in the mouse brain (4). Indeed, we confirmed GFP expression in CD206^+^ choroid plexus macrophages and perivascular macrophages from both genotypes (Supplemental Figure 12), indicating an adult (definitive) hematopoietic contribution to this population (4). Interestingly, GFP^+^ plaque-associated cells lose expression of *Ccr2* (which is observed in GFP^+^ cells in the perivascular spaces, meninges, and choroid plexus; Supplemental Figure 13) and express *Hexb* (Supplemental Figure 14), a microglia-enriched transcript (30), which is also observed to be expressed in engrafted monocyte-derived macrophages in the brain parenchyma in the setting of experimental microglial ablation (31). Moreover, GFP^+^ cells in the brain parenchyma do not express *Tmem119* (Supplemental Figure 15), a microglia-enriched transcript that is detectable in APP/PS1 brains (Supplemental Figure 15) but downregulated in the 5XFAD model of accelerated amyloid pathogenesis (30). Taken together, these data suggest that peripheral monocyte–derived amyloid plaque–associated cells are a unique cellular population.

**Figure 4:**
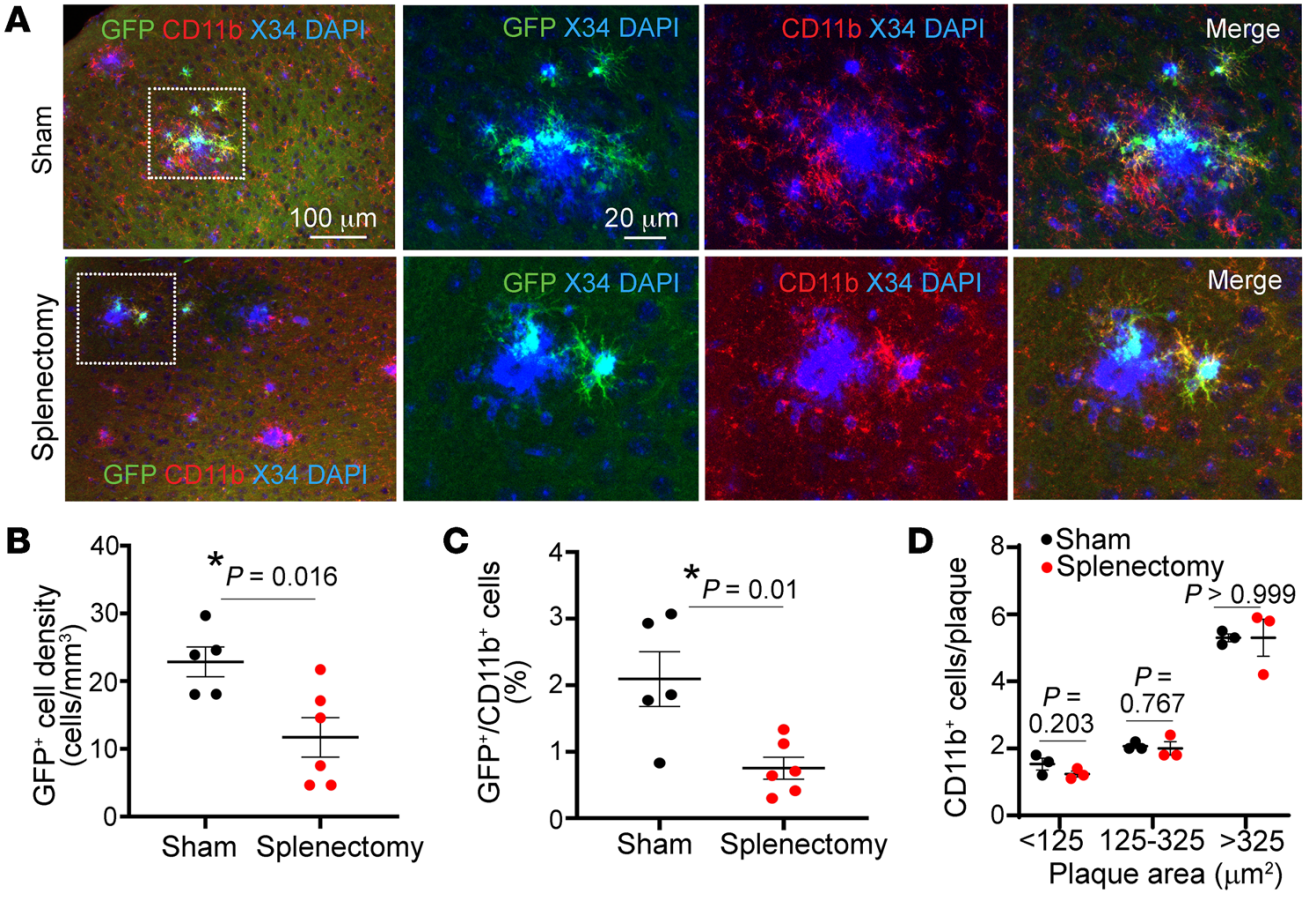
Splenectomy results in reduced monocyte-derived periplaque macrophages. (**A**) Representative cortical sections from APP/PS1^mTmG;Flt3-Cre^ mice subjected to splenectomy at 5.5 months of age and sacrificed at 9.5 months of age, demonstrating fewer GFP^+^CD11b^+^ cells next to amyloid plaques stained with X-34. Areas boxed by dotted lines are shown at higher magnification on the right. (**B**) Density of GFP^+^ cells in the brain parenchyma and (**C**) quantitation of GFP^+^ cells as a fraction of CD11b^+^ plaque-associated cells in mice from **A**. (**D**) Abundance of CD11b^+^ cells adjacent to amyloid plaques stratified by size in mice from **A**. *n =* 6 after splenectomy; *n =* 5 after sham procedure. *P < 0.05, *t* test.

Previous studies have demonstrated that the spleen contributes to definitive hematopoiesis in mice (32) and acts as a reservoir of monocytes for sustained release into the circulation with ongoing inflammatory stimuli (33). Indeed, splenic hypertrophy was observed in a triple-transgenic AD model (34). Furthermore, in the post–myocardial infarction state, the spleen was demonstrated to be a major source of circulating Ly6c^hi^ monocytes (35, 36), a monocyte subset with high potential of migration to tissues (37). Indeed, splenectomy in the face of chronic stress attenuates inflammatory cell infiltration in the failing heart (38), in dystrophic muscle (39), after stroke (40), and in abdominal aortic aneurysms (41). To determine the functional contribution of circulating monocytes to amyloid plaque pathogenesis, we performed splenectomy on male APP/PS1^mTmG;Flt3-Cre^ mice. Four months after splenectomy, we observed an approximately 50% reduction in GFP^+^ cells adjacent to the amyloid plaques (Figure 4, A–C), suggesting that elimination of the splenic reservoir reduced the abundance of plaque-associated macrophages of definitive hematopoiesis origin. This result also suggests that the GFP^+^ macrophages in the brain parenchyma of APP/PS1 mice were peripherally derived macrophages, rather than locally proliferating GFP^+^ macrophages. Notably, splenectomy did not alter the total population of periplaque CD11b^+^ cells, irrespective of the plaque size (Figure 4D).

**Figure 5:**
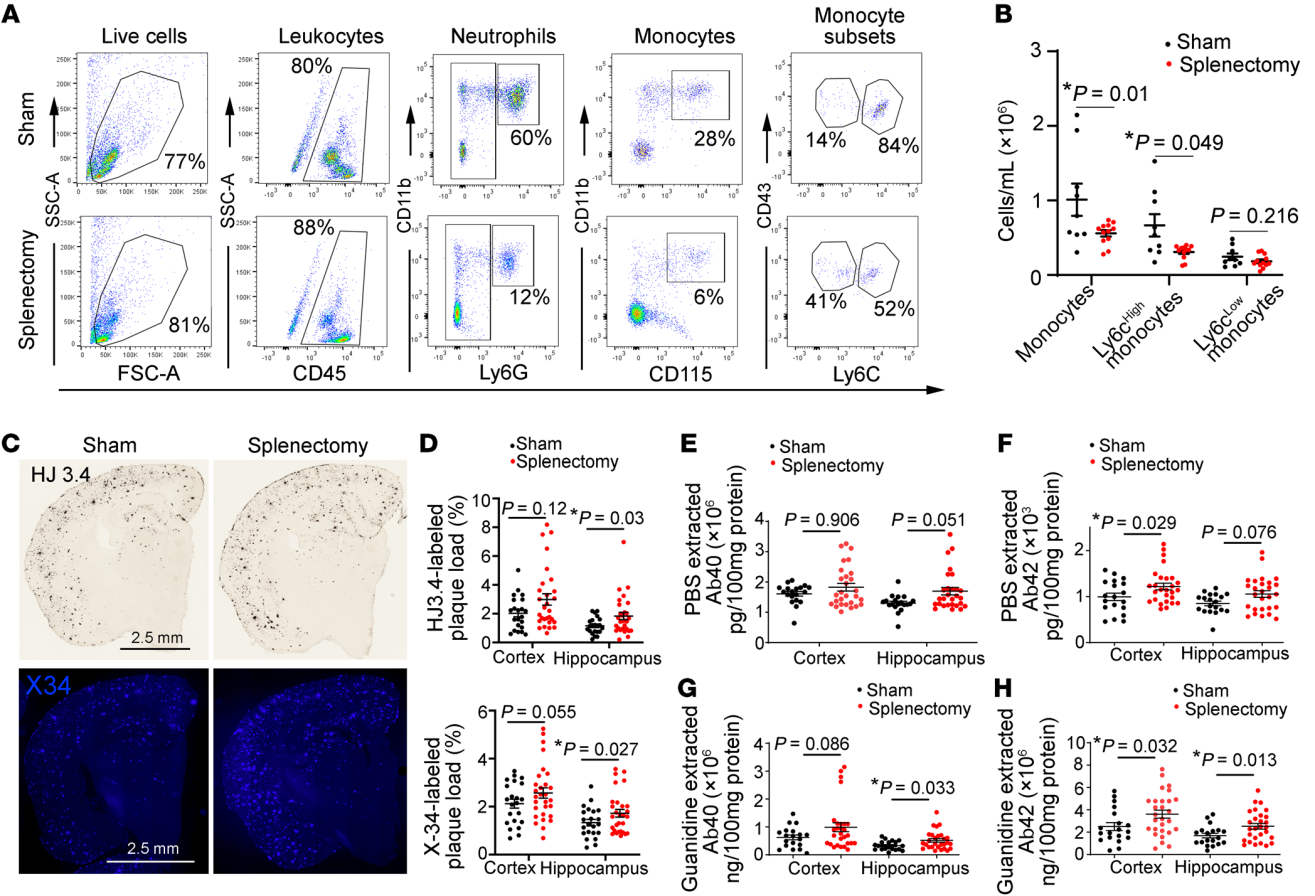
Splenectomy reduces circulating myeloid cells and worsens amyloid pathology. (**A** and **B**) Flow cytometric phenotyping strategy A (after doublet exclusion was performed) for assessment of total white blood cells, monocytes (total, and separately as Ly6c^hi^ and Ly6c^lo^), and neutrophils in APP/PS1 female mice 4 months after splenectomy compared with matched sham-operated controls. Grouped data for total monocytes and Ly6c^hi^ and Ly6c^lo^ monocytes is shown in **B**. *n =* 13 splenectomy; *n =* 9 sham. (**C**) Representative images of HJ3.4-stained (anti-Aβ antibody) (top) and X-34–stained compact amyloid plaques (bottom), (**D**) with quantitation of plaque load in the cortex and hippocampus by HJ3.4 staining (top) and X-34 staining (bottom) in female APP/PS1 mice after splenectomy. *n =* 22 after splenectomy; *n =* 29 after sham procedure. Scale bar: 2.5 mm. (**E**–**H**) Quantitative assessment of Aβ40 and Aβ42 levels in PBS-extracted and guanidine-extracted brain tissue from APP/PS1 mice subjected to splenectomy, as in **D**. *n =* 26 after splenectomy; *n =* 21 after sham procedure. *P < 0.05, (**B**) *t* test, except for comparison of Ly6c^hi^ monocytes by Wilcoxon’s rank-sum test; (**D**) *t* test, except for comparison of X-34–stained plaques in hippocampus where a Wilcoxon’s rank-sum test was employed; (**E**) Wilcoxon’s rank-sum test; and (**F**–**H**) *t* test, except for comparison of Aβ42 levels in hippocampus in **F**, where Wilcoxon’s rank-sum test was employed.

Given that Flt3-Cre is expressed via the Y chromosome (29), which precludes direct examination of the fate-mapping reporter system in females, we focused our analyses on evaluation of effects of splenectomy on amyloid pathogenesis in female mice. Splenectomy resulted in a significant reduction in circulating Ly6c^hi^ monocytes (Figure 5, A and B) and neutrophils (2,79,501 ± 21,119 cells/mL [mean ± SEM] after splenectomy vs. 23,71,722 ± 5,40,631 cells/mL [mean ± SEM] in sham; *n =* 9–12; *P =* 0.0002 by *t* test), which were not recruited into the AD brain parenchyma (as shown in Supplemental Figure 8). Notably, splenectomy led to a modest but significant increase in HJ3.4–labeled amyloid plaque as well as in X-34–stained compact plaque in the hippocampus (Figure 5, C and D), as compared with that in sham controls. Splenectomy also resulted in significant increases in soluble and guanidine-extractable Aβ42 in the cortex and guanidine-extractable Aβ40 and Aβ42 in the hippocampus (Figure 5, E–H). Taken together, these data point to a functional role for circulating monocyte-derived macrophages in limiting amyloid plaque pathogenesis.

## Discussion

Our data provide strong evidence that peripheral monocytes enter the AD brain and specifically home in to amyloid plaques. The Flt3-based fate-mapping strategy overcomes the limitations of prior approaches by the continuously renewed expression of the reporter in circulating monocytes in a mouse model with slower plaque growth that mimics the clinical evolution of pathology in AD. This may explain why these cells were missed in recently reported fate-mapping studies that selectively labeled cells with limited life spans (21). While the contribution of peripheral monocyte–derived macrophages to the total activated microglial/macrophage population may appear small (Table 1), it is likely that recruitment is a dynamic and ongoing process, evidenced by the increased appearance of peripheral cells at entry points to the brain (Figures 1 and 2 and Supplemental Figure 4). Moreover, unlike dysfunctional microglia in aged APP/PS1 mice, which can only renew through in situ proliferation, peripherally derived macrophages have a theoretical advantage of taking up amyloid plaque and, if toxicity occurs, being replaced by newly formed and fully functional macrophages.

Importantly, these peripheral monocytes migrate selectively to amyloid plaques and take on microglial markers, suggesting recruitment of newly derived macrophages (31) to reinforce microglial phagocytosis of amyloid material by virtue of being naive to prior amyloid exposure. Indeed, our observations indicate that, while splenectomy does not eliminate the circulating monocytes (with an ~53% reduction in circulating Ly6C^hi^ monocytes (Figure 5, A and B), it induces a significant reduction in plaque-associated monocyte-derived macrophages of approximately 50%, with increased amyloid plaque pathogenesis over a period of 4 months (Figure 5, C–H). Given that splenectomy does not alter the total number of activated (CD11b^+^) macrophages associated with amyloid plaques (Figure 4D), these effects of splenectomy are likely transduced by reduced monocyte recruitment into the brain parenchyma and are consistent with the enhanced ability of newly recruited monocytes to degrade amyloid material as compared with the resident microglia exposed to progressive amyloid pathology.

While our data do not address the potential for pleiotropic mechanisms whereby splenectomy affects AD pathogenesis (42), these observations point to the spleen as a potential reservoir for sustained recruitment of these cells to the amyloid plaques, akin to its role in other pathologic states (33, 38, 39, 41). Given multiple lines of evidence pointing to a role of microglial dysfunction in progression of AD pathogenesis, harnessing the role of peripheral monocytes in limiting amyloid plaque pathogenesis has potential therapeutic implications. Future studies will be required to assess the kinetics of peripheral monocyte recruitment, survival, and proliferation in the AD brain parenchyma to comprehensively assess the therapeutic potential of targeting this more accessible cellular population (as compared with dysfunctional microglia confined within the brain) in prevention and treatment of AD.

## Methods

### Fate-mapping studies.

Male APP/PS1 mice [B6;C3-Tg(APPswe,PSEN1dE9)85Dbo/Mmjax, ref. 43; The Jackson Laboratory, MMRRC stock no. 34928, maintained as C57BL/6 × C3H strain] carrying the mTomato-lox-STOP-GFP cassette in the *Rosa26* locus (The Jackson Laboratory, stock no. 007676) (44) and *Flt3* promoter–driven Cre transgene (as it is expressed on the Y chromosome, ref. 29; RosamTmG:*Flt*3-Cre; both mice were of the C57BL/6 strain) were generated. Littermate male mice without the APP/PS1 transgene were employed as controls. Cre-mediated excision of the mTomato cassette permits expression of GFP exclusively in monocytes and is not observed in microglia, which are derived from primitive hematopoietic precursors in the yolk sac. Mice were sacrificed beginning at 6 months of age. After thorough perfusion with PBS to remove circulating blood cells, one brain hemisphere was homogenized; mononuclear cells were isolated on a percoll gradient, as previously described (45); and live cells were evaluated using flow cytometry. The other hemisphere was used for histology.

### Studies with splenectomy.

Splenectomy and sham surgery was performed in 4.5-month-old female APP/PS1 mice (sham, *n =* 22; splenectomy, *n =* 29), following previously described surgical technique (46). The animals were randomly assigned to the splenectomy procedure. Animals were anesthetized with induction of 3%–4% isoflurane and maintained at 2%. A left-side dorsal incision was made lateral to the spine, and the abdominal cavity was entered. The splenic blood vessels were ligated, and the spleen was removed by transecting the vessels just distal to the ligature. The skin incision was closed with wound clips. Sham surgery was performed without ligating blood vessels and removing the spleen. Following surgery, mice were aged to 8.5 months (±0.5 months); blood was collected for flow cytometry, and brains were harvested for histological analysis. Peripheral blood was used to perform flow cytometry experiments. Mice were cheek bled via the facial vein, and red blood cells were lysed in lysis buffer (BD Pharmlyse). Nucleated peripheral blood cells were then washed once in PBS and incubated with appropriate antibodies in PBS containing 0.2% BSA on ice for 40 minutes and analyzed on a FACScan flow cytometer (BD). Fluorescence data were analyzed by FlowJo, BD FACS Diva analysis software. A mixture of the following mixture was used for flow cytometry: FITC anti-mouse CD11b (Bioscience); APC anti-mouse CD115 (Biolegend); APC-cy7 anti-mouse Ly6G (Biolegend); PE anti-mouse CD43 (Bioscience); PerCP-cy5.5 anti-mouse Ly6c (Bioscience); and Pacific Blue anti-mouse CD45 (Biolegend). In a separate cohort, splenectomy was performed in male APP/PS1 mice carrying the mTomato-loxp-STOP-GFP cassette in the Rosa26 locus and Flt3 promoter–driven Cre transgene at approximately 5.5 months of age, and brains harvested at 9.5 months of age to assess for presence of GFP^+^ cells adjacent to plaques.

### Flow cytometry.

Mice were cheek bled via the facial vein in 20 μL EDTA (100 mM), and red blood cells were lysed in lysis buffer (BD Pharmlyse). Nucleated peripheral blood cells were then washed once in PBS and incubated with appropriate antibodies in PBS containing 0.2% BSA on ice for 20 minutes, and cells were analyzed on a Fortessa or LSR II (BD). Blood monocytes were stained by Pacific Blue anti-CD45 (30-F11, Biolegend), APCCy7anti-CD11b (M1/70, Biolegend), APC anti-CD115(AFS98, eBioscience), and PerCP anti-Ly6C (HK1.4, Biolegend). Brain parenchymal cells were stained by Pacific Blue anti-CD45, APCCy7 anti-CD11b, and PECy7 anti-Ly6G (1A8, Biolegend). Flow cytometric data were analyzed by FlowJo, BD FACS Diva analysis software.

### Immunohistochemistry.

Brains were perfused, removed, and divided into hemispheres. One hemisphere was fixed for 24 hours in 4% paraformaldehyde fixative in 0.1 M phosphate buffer (PB) (pH 7.4) and then transferred to a solution containing 30% sucrose in 0.1 M PB overnight. The tissue was then sectioned (30 μm) and immunostained using antibodies delineated below. Brain sections were permeabilized and blocked with 0.3% Triton X-100/ 3% dry milk in 0.01 M PBS for 30 minutes followed by incubation with primary antibodies overnight at 4°C and fluorescently labeled secondary antibodies at 37°C for 1 hour. Primary and secondary antibodies employed are shown in Supplemental Table 2. GFP-tagged cells in the cortex, immunostained with Iba1 or CD11b, were counted in 6 equally spaced sections and expressed as a percentage of the total number of Iba1 and CD11b cells per section. Slides were mounted and examined with a Nikon A1Rsi Confocal Microscope (Washington University Center for Cellular Imaging). In control sections, the primary antibody was substituted by 3% dry milk in 0.01 M PBS. GFP-tagged cells in the cortex (which are near the amyloid plaques) were immunostained with Iba1, CD11b, or CD68; counted in 3 equally spaced sections (180 μm apart), and expressed as a percentage of the total number of Iba1-, CD11b-, and CD68-expressing cells per section. For analysis of GFP-tagged cells in the choroid plexus, meningeal, and perivascular structures, GFP^+^ cells were expressed as a percentage of total CD45- or CD68-expressing cells.

### Assessment of amyloid plaques.

For X-34 staining, brain slices were mounted on glass slides. Tissue was permeabilized with 0.25% Triton for 30 minutes and stained with X-34 dissolved in a solution of 40% ethanol in water, pH 10, for 20 minutes. Tissue was then rinsed in distilled water and mounted. For amyloid staining, sections were permeabilized with 0.3% Tween-20 in Tris-buffered saline (TBS-T20) for 10 minutes, and endogenous peroxidase activity was quenched by a 10-minute treatment with 0.3% H_2_O_2_ solution in TBS. Tissue was washed with TBS, blocked with 3% dry milk in TBS-T20 for 1 hour, and incubated with anti-Aβ antibody (HJ3.4, 1:1000) antibody overnight. A fresh solution of streptavidin and horseradish peroxidase–conjugated biotin (1:400, Vector Laboratories) was applied to tissue for 90 minutes, followed by 0.025% 3-3′-diaminobenzidine tetrachloride in 0.25% NiCl and 0.05% H_2_O_2_ for 10 minutes. The plaque load in 6 equally spaced sections (180 μm apart) per mouse were analyzed and expressed as a percentage area of the cortex.

### In situ hybridization by RNAscope technology.

RNAscope Multiplex Fluorescent Assay was combined with an immunofluorescence technique to detect transcripts for *Tmem119*, *Ccr2*, and *Hexb*, in concert with imaging amyloid plaques (HJ3.4 antibody) and microglia (Iba1 antibody) in the mouse brain tissue. The RNA scope probe for the *Ccr2* gene is *Mus musculus* chemokine (C-C motif) receptor 2 (Ccr2) mRNA (catalog 501681, Advanced Cell Diagnostics Inc.). The RNA scope probe for the *Tmem119* gene is *Mus musculus* transmembrane protein 119 mRNA (catalog 472901-C2, Advanced Cell Diagnostics Inc.). The RNA scope probe for the *Hexb* gene is *Mus musculus* hexosaminidase B (Hexb) mRNA (catalog 314231-C1, Advanced Cell Diagnostics Inc.). For fluorescent detection of mRNA signals, the fluorophores Opal 570 (1:2000) (Opal570 Reagent Pack, PN FP1488001KT, Akoya Biosciences) and Opal 520 (1:2000) (PN FP1487001KT, Akoya Biosciences) were used. Based on the manufacturer’s fixed frozen tissue protocol, mouse brain cryostat sections (30 μm thickness) postfixation, were processed for target retrieval, protease treatment, hybridization with target probes, preamplifier, amplifier, and Opal dye incubation (RNAscope Multiples Fluorescent Reagent Kitv2 Assay, ACD). Briefly, after fixation in 4% paraformaldehyde in 0.1 M PB for 40 minutes, brain sections were incubated in citrate buffer (10 nmol/L, pH 6) maintained at a boiling temperature (100°C–103°C) using a hot plate for 10 minutes, rinsed in deionized water, and immediately treated with RNAscope protease III at 40°C for 30 minutes. Hybridization with probes was performed for 2 hours at 40°C, followed by serial RNAscope Multiplex FFLv2 AMP steps at 40°C for 30 minutes, 30 minutes, and 15 minutes, respectively. Opal Dye fluorophore was applied on the sections at 40°C for 30 minutes. After RNAscope staining, the sections were processed for fluorescence immunohistochemistry staining. The RNAscope 3-plex Negative control probe and the RNAscope 3-plex positive control probe were used to assess tissue RNA integrity, assay procedure, and background signals. The Microscopic Imaging Nikon A1Rsi Confocal Microscope (Nikon) was used for imaging and analysis. Analysis of GFP^+^CCR2^+^ cells in the choroid plexus and meningeal and perivascular structures was performed in 4 sections/mouse and is reported per unit area.

### Biochemical assessment of Aβ levels.

To measure Aβ, dissected cortices or hippocampi were homogenized in PBS and then in 5 M guanidine in TBS, pH 8.0 (to extract fibrillar and membrane bound Aβ). Aβ_x–40_ and Aβ_x–42_ were assessed using mouse monoclonal capture antibodies HJ2 (anti-Aβ35–40) and HJ7.4 (anti-Aβ37–42), respectively, and a biotinylated central domain antibody, HJ5.1 (anti-Aβ13–28), was used as the detecting antibody, followed by streptavidin-poly-HRP-40 (Fitzgerald Industries), as previously described (47). All ELISA assays were developed using Super Slow ELISA TMB (MilliporeSigma), and absorbance was read on a Bio-Tek Epoch plate reader at 650 nm. Standard curves were generated from synthetic human Aβ_1–40_ or Aβ_1–42_ peptides (American Peptide).

### Statistics.

Results are expressed as mean ± SEM. Assumptions of normality were examined by visual display and Shapiro-Wilk test. Log transformation to natural base was applied for data that were not normally distributed. Statistical differences were assessed with the unpaired 2-tailed Student’s *t* test for 2 experimental groups (Prism, version 5.2) for data that were normally distributed. For data that failed normality testing, Wilcoxon’s rank-sum test was employed. For data sets with a small sample size (Figure 2D), a permutation test was used to compare the mean between 2 groups (independence_test () in R package coin) (48). A 2-tailed *P* value of less than 0.05 was considered statistically significant.

### Study approval.

All animal studies were approved by the IACUC at Washington University School of Medicine.

## Author contributions

PY and KWK performed experiments; acquired, analyzed, and collated the data; and assisted with manuscript preparation. QX, XM, DRR, LRC, and HL performed experiments and acquired and analyzed the data. YY analyzed the data. GJR, SE, JML, and AD designed the study and wrote the manuscript. All authors read and approved the final manuscript.

## Supplementary Material

Supplemental data

## Figures and Tables

**Table 1:**
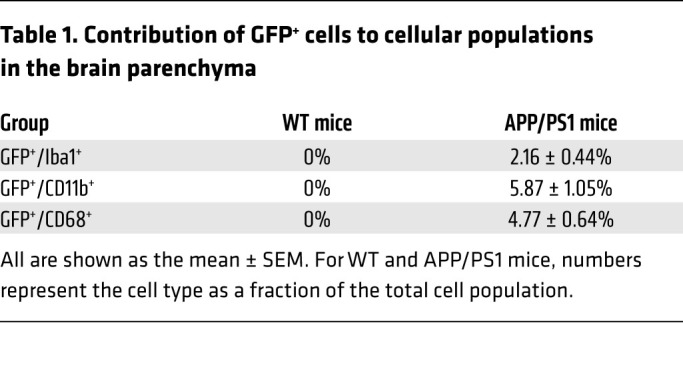
Contribution of GFP^+^ cells to cellular populations in the brain parenchyma

## Version 1 05/05/2022

In-Press Preview

## Version 2 06/01/2022

Electronic publication
