# Supplementary material for: Peripheral monocyte–derived cells counter amyloid plaque pathogenesis in a mouse model of Alzheimer’s disease
Source: J Clin Invest. 2022 Jun 1;132(11):e152565. doi: 10.1172/JCI152565 (PMC9151689; doi:10.1172/JCI152565)
Supplement: Supplemental data [file jci-132-152565-s179.pdf]

Supplementary Figure Legends:

Supplementary Figure S1: Fate mapping strategy demonstrates robust expression of GFP by *Flt3* promoter-driven Cre excision of the STOP codon in mice carrying the mTmG cassette in circulating monocytes in the blood. A, B) Flow cytometric phenotyping strategy (A, after doublet exclusion was performed) to quantitate GFP expression in monocytes (B) in APP/PS1mice and wild-type controls older than 10 months of age. N=6 APP/PS1 and N=4 wild-type. P value depicted for B is by unpaired two-tailed t-test.

Supplementary Figure S2: Fate mapping strategy demonstrates a strong trend towards increased expression of GFP+ microglia-like cells in the brain. A) Flow-cytometric phenotyping strategy (25,000 cells/mice) on brain parenchymal cells pre-gated on CD45+ cells from $WT^{mTmG;Flt3\text{-}Cre}$ (N=4) and $APP/PS1^{mTmG;Flt3\text{-}Cre}$ (N=7) mice between 10-17 months of age. **B)** Relative expression of GFP+ cells in the CD45+ cells in WT (n=4) and APP/PS1 (n=7) mice carrying the fate mapping cassette. **C)** Relative expression of GFP+ cells in the $CD45^{high}Ly6G^{low}Cd11b+$ cell population. **D)** Relative expression of GFP+ cells in the $CD45^{int}Ly6G^{low}Cd11b+$ cell population. For B, C and D; the P values are by two-tailed t-test. Data from n=2 APP/PS1 mice and n=1 WT mice were excluded due to technical limitations.

Supplementary Figure S3: GFP+ cells co-localize with macrophage markers in the perivascular space and meninges. A. Co-localization of GFP+ and Iba1+ cell within the perivascular space delineated by immunostaining for lectin. **B**. Co-localization of GFP+ and ER-TR7 (fibroblast-specific antigen) in the meninges.

Supplementary Figure S4: Fate mapping strategy demonstrates increased contribution of definitive hematopoiesis-derived cells to the choroid plexus and perivascular space leukocyte population. A-F) Representative images demonstrating GFP expression in CD45+ cells in the choroid plexus in

WT$^{mTmG;Flt3\text{-}Cre}$ and APP/PS1$^{mTmG;Flt3\text{-}Cre}$ mice between 10-17 months of age in the choroid plexus (A), perivascular space (D) and meninges (E); with quantitation of GFP expression in this cell population in the respective populations (B, D, and F). N=7-8 mice/group. P values depicted are by two-tailed t-test. * denotes $P<0.05$.

Supplementary Figure S5: GFP+ cells do not express astrocytic or neuronal markers. A, B) Cortical sections from APP/PS1 brain demonstrating lack of co-localization of GFP (arrow) with GFAP (astrocyte marker, A), and NeuN (neuronal marker, B). N=8 mice were examined.

Supplementary Figure S6: GFP expressing B cells are detected in the choroid plexus but not in the brain parenchyma. Expression of CD45r (a B-cell expressed antigen) was detected in a fraction of GFP+cells in the choroid plexus of both wild-type (*top* panel, arrow) and APP/PS1 mice carrying the mTmG:Flt3Cre cassette (*middle* panel, arrow), but not in the plaque-associated GFP+ cells in APP/PS1 mice (*bottom* panel, arrow). N=6 mice were examined in each group.

Supplementary Figure S7: GFP expressing T cells are detected in the choroid plexus but not in the brain parenchyma. Expression of CD3e (a T-cell expressed antigen) was detected in a fraction of GFP+cells in the choroid plexus of both wild-type (*top* panel, arrow) and APP/PS1 mice carrying the mTmG:Flt3Cre cassette (*middle* panel, arrow), but not in the plaque-associated GFP+ cells in APP/PS1 mice (*bottom* panel, arrow). N=6 mice were examined in each group.

Supplementary Figure S8: Neutrophils are not detected in the brain parenchyma of mice. Expression of Ly6G (a neutrophil-expressed antigen) was detected in the choroid plexus from APP/PS1 mice carrying the mTmG:Flt3Cre cassette (2nd panel from top, arrow) but not in the brain parenchyma (3rd panel from top) or in wild-type (top panel). A section from spleen (*bottom* panel, arrow) is shown as positive control. N=6 mice were examined in each group.

Supplementary Figure S9: NK cells are not detected in the brain parenchyma of mice. Expression of NK1 .1 (a NK cell-expressed antigen) was detected in the choroid plexues of APP/PS1 mice carrying the mTmG:Flt3Cre cassette (2nd panel from top, arrow) but not in the brain parenchyma (3rd panel from top) or in choroid plexus from wild type mice (top panel). A section from spleen (*bottom* panel, arrow) is shown as positive control. N=6 mice were examined in each group.

Supplementary Figure S10. FLT3 expressing cells are not detected in the brains of mice. A) Expression of FLT3 protein was not detected either aged (10 month old) wild-type (*top* panel, arrow) and APP/PS1 mice carrying the mTmG:*Flt3*Cre cassette (*2nd* panel from top, arrow). N=6 mice were examined in each group. **B**) Flt3 protein expression was not detected by immunohistochemistry in wild type brains at the indicated ages. **C)** A section from the bone marrow (arrow) is shown as positive control.

Supplementary Figure S11: GFP-tagged peripheral myeloid population-derived cells express macrophage and microglial markers. Representative cortical sections from aged APP/PS1$^{mTmG;Flt3\text{-}Cre}$ mice demonstrating that GFP+ cells seen adjacent to X-34 stained amyloid plaques (outlined) in the APP/PS1 transgenic mice express CD45 (*top panel*), TREM2 (*middle panel*) but not CD206 (*bottom panel*); see inset for cells pointed to with arrows. Arrowheads in D indicate CD206+ cells in the meninges.

Supplementary Figure S12: GFP-positive CD206 expressing cells are detected in the choroid plexus and perivascular space, but not in the brain parenchyma. Expression of CD206 was detected in a fraction of GFP+cells in the choroid plexus of both wild-type and APP/PS1 mice carrying the mTmG:*Flt3*Cre cassette (see arrows, *top* and *middle* panel) and in the perivascular space (see arrowhead for GFP+ cell in the perivascular space, *bottom* panel) but not in the plaque-associated GFP+ cells in APP/PS1 mice (see arrow, *bottom* panel). N=6 mice were examined in each group.

Supplementary Figure S13: GFP+ cells express *Ccr2* in the perivascular space, meninges and choroid plexus, but not in the brain parenchyma. A-I. In situ hybridization was performed to detect *Ccr2* transcript expression in APP/PS1 brains harboring the fate-mapping cassette. *Ccr2* expression (red) is observed in GFP+ perivascular macrophages (seen in A, B, F), meningeal macrophages (E), and choroid plexus macrophages (G), but not in GFP+ peri-plaque macrophages (C, D). Spleen tissue from a *Ccr2* null mouse (I) and wild type (H) is shown as a control.

Supplementary Figure S14: **GFP+ cells express *Hexb* in the brain parenchyma, but not in the perivascular space, meninges and choroid plexus. A-J.** In situ hybridization was performed to detect Hexb transcript expression in APP/PS1 brains harboring the fate-mapping cassette. *Hexb* expression is observed in endogenous microglia and GFP+ peri-plaque macrophages (A-D) but not observed in GFP+ perivascular macrophages (seen in E), meningeal macrophages (F), and choroid plexus macrophages (G). *Hexb* expressing cells also express iba1+ (H, I, L). Dotted lines indicate areas shown in magnified images in figure panels A-G.

Supplementary Figure S15: GFP+ cells do not express TMEM119, a microglia-specific transcript. In situ hybridization was performed to detect TMEM119 transcript expression in APP/PS1 brains harboring the fate-mapping cassette. TMEM119 expression is observed in endogenous microglia but not in GFP+ peri-plaque macrophages.

Table S1: Table depicting quantitation of GFP+ cells as a % of Cd11b+ cells in the brain parenchyma of APP/PS1 mice at the indicated ages.

ID	age	% GFP/CD11b
M1	6m	0.00
M2	6m	0.00
M3	6m	0.00
M4	10m	8.34
M5	10m	9.17
M6	10m	9.04
M7	10m	2.52
M8	10m	1.02
M9	14m	5.72
M10	15m	5.54
M11	17m	5.58

Quantitative data from 10 month and older mice from this table are summarized in Table 1.

Table S2: Sources and concentrations of antibodies employed for immuno-histochemistry.

Antigen	Concentration of primary antibody	Source of primary antibody	Concentration of secondary antibody	Source of secondary antibody
Iba1	1:1000	rabbit anti iba1 Wako Pure Chemical Industries, Ltd. cat# 019-19741	1:1800	anti-rabbit Alex49 Life science
Aβ (HJ3.4)	1:1000	Biotinylated antibody, gift from Dr. David M Holtzman (1)	N/A	Developed with PK4001 Vectastain ABC kit
CD11b	1:200	rat anti-CD11b Cat # 55082 BD Pharminigen	1:1800	Ant- rat Alexa 649 Life science
OX42	1:500	muse anti-OX42 Abcam Ab1211	1:1800	anti-mouse Alex49 Life science
CD68	1:100	rat anti CD68 eBioscience cat#14-0681-81	1:1800	Ant- rat Alexa 649 Life science
CD45	1:1000	rat anti-CD45 Cat # 550539 BD Pharminigen	1:1800	Ant- rat Alexa 649 Life science
TREM2	1:400	Sheep anti TREM2 R&D cat# AF1729	1:400; 1:1800	Biontinylted anti sheep IgG, anti-biotin Streptavidin 649
CD11c	1:100	Hamster anti CD11c Biotech Cat #117301	1:400; 1:1800	Biontinylted anti hamster IgG, anti-biotin Streptavidin 649
MHC II	1:100	Rat anti MHC II (I-AVI-E) APC (=cy5) cat# 17-5321-82 eBioscience	N/A	N/A
CD206	1:200	Rat anti CD206 AdD Serotec MCA2235T	1:800	Ant- rat Alexa 649 Life science
CD45r	1:200	Rat Anti-Human/Mouse CD45R (B220; eBioscience)	1:5000	anti-rat Cy5, Life science
CD3e	1:200	anti-mouse CD3e clone 145-2c11 (553058, BD Pharmingen)	1:500	anti-hamster Cy5, Life science
Flt3	1:400	Polyclonal Rabbit anti-Mouse CD135 / FLT3 Antibody (Biotin, aa335-544)		
GFP	1:500	Goat anti-GFP (Rockland antibodies)	1:800	Anti-goat Alexa488 (Life Sciences)
Lectin	1:400	Biotinylated lycopersicon ESCULENTUM (Tomato) Lectin Certor B-1175		
ER-TR7	1:500	Rat anti Fibroblast Antibody (ER-TR7)		

		NOVUS Biologicals NB 100-64932SS		

Supplementary References

1. Kim J, Castellano JM, Jiang H, Basak JM, Parsadanian M, Pham V, et al. Overexpression of low-density lipoprotein receptor in the brain markedly inhibits amyloid deposition and increases extracellular A beta clearance. *Neuron.* 2009;64(5):632-44.

Figure S1

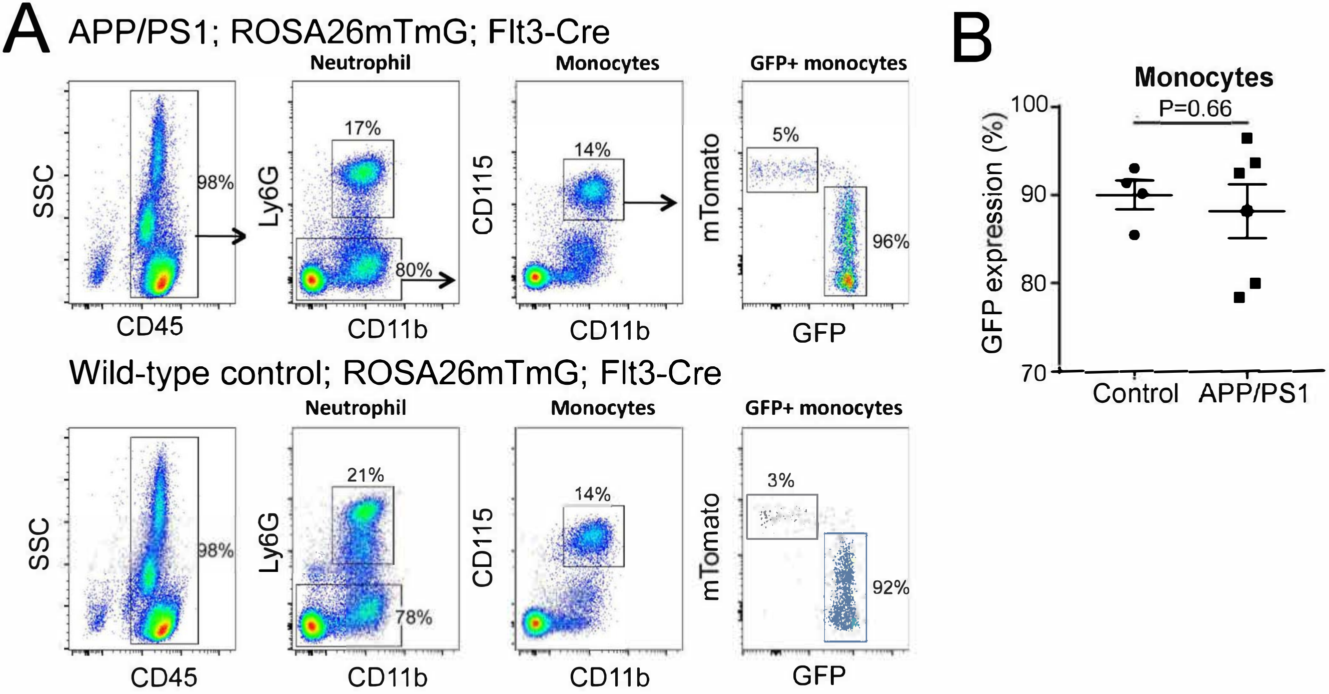

Supplementary Fig. S1 A-C: Fate mapping strategy demonstrates robust expression of GFP by *Flt3* promoter-driven Cre-mediated excision of the STOP codon in mice carrying the mTmG cassette in circulating monocytes in the blood. A, B) Flow cytometric strategy (A) to quantitate GFP expression in monocytes (B) in APP/PS1 mice and wild-type controls older than 10 months of age. N=6 APP/PS1 and n=4 wild type.

Figure S2

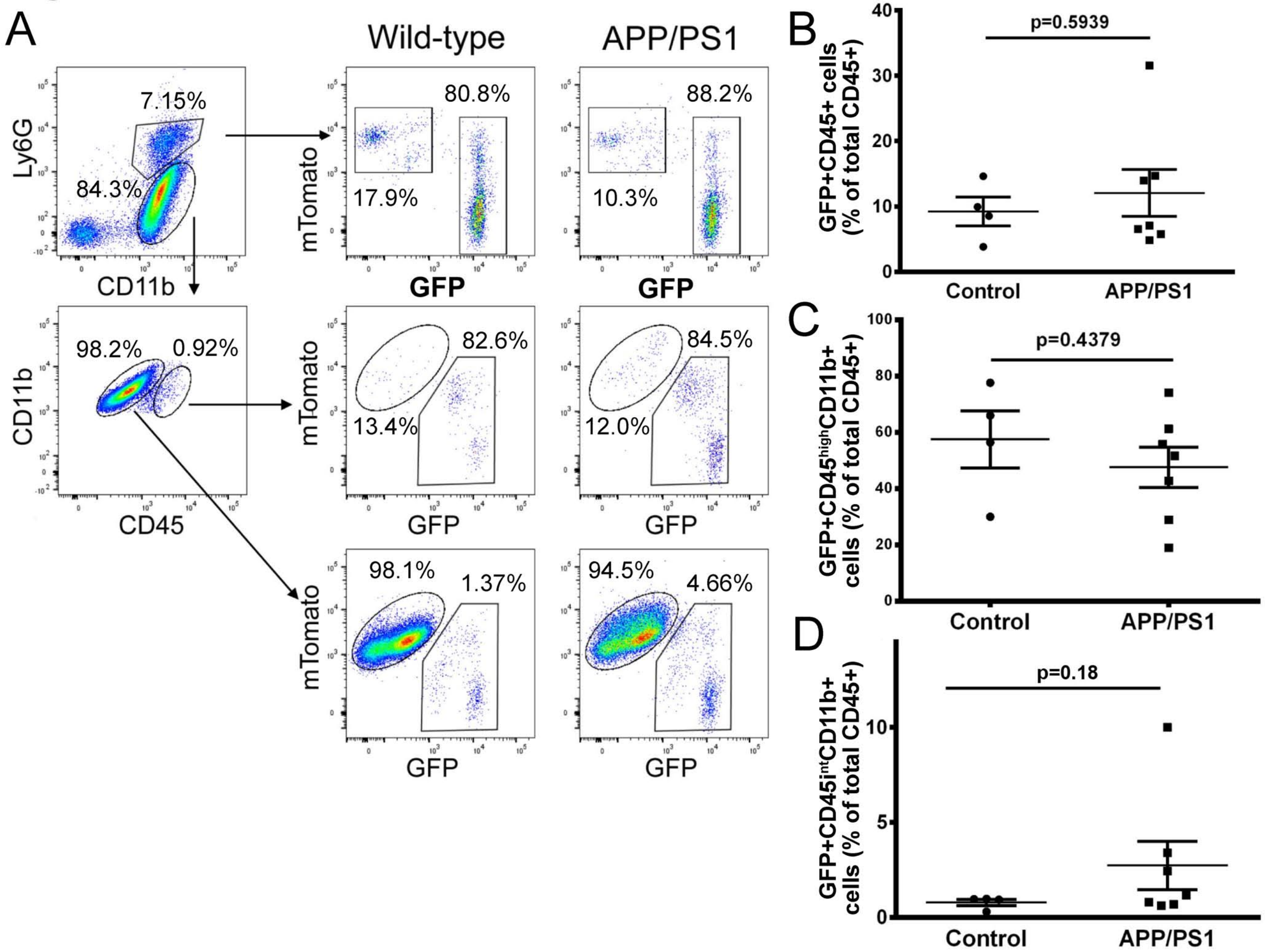

Supplementary Figure S2: Fate mapping strategy demonstrates a strong trend towards increased expression of GFP+ cells in the brain. A) Flow-cytometric phenotyping strategy (25,000 cells/mice) on brain parenchymal cells pre-gated on CD45+ cells from WT$^{mTmG;Flt3\text{-}Cre}$ (N=4) and APP/PS1$^{mTmG;Flt3\text{-}Cre}$ (N=7) mice between 10-17 months of age. **B)** Relative expression of GFP+ cells in the CD45+ cells in WT (n=4) and APP/PS1 (n=7) mice carrying the fate mapping cassette. **C)** Relative expression of GFP+ cells in the CD45^{high}Ly6G^{low}Cd11b+ cell population. **D)** Relative expression of GFP+ cells in the CD45^{int}Ly6Gl^{ow}Cd11b+ cell population. For B, C and D; the P values are by two-tailed t-test. Data from n=2 APP/PS1 mice and n=1 WT mice were excluded due to technical limitations.

Figure S3

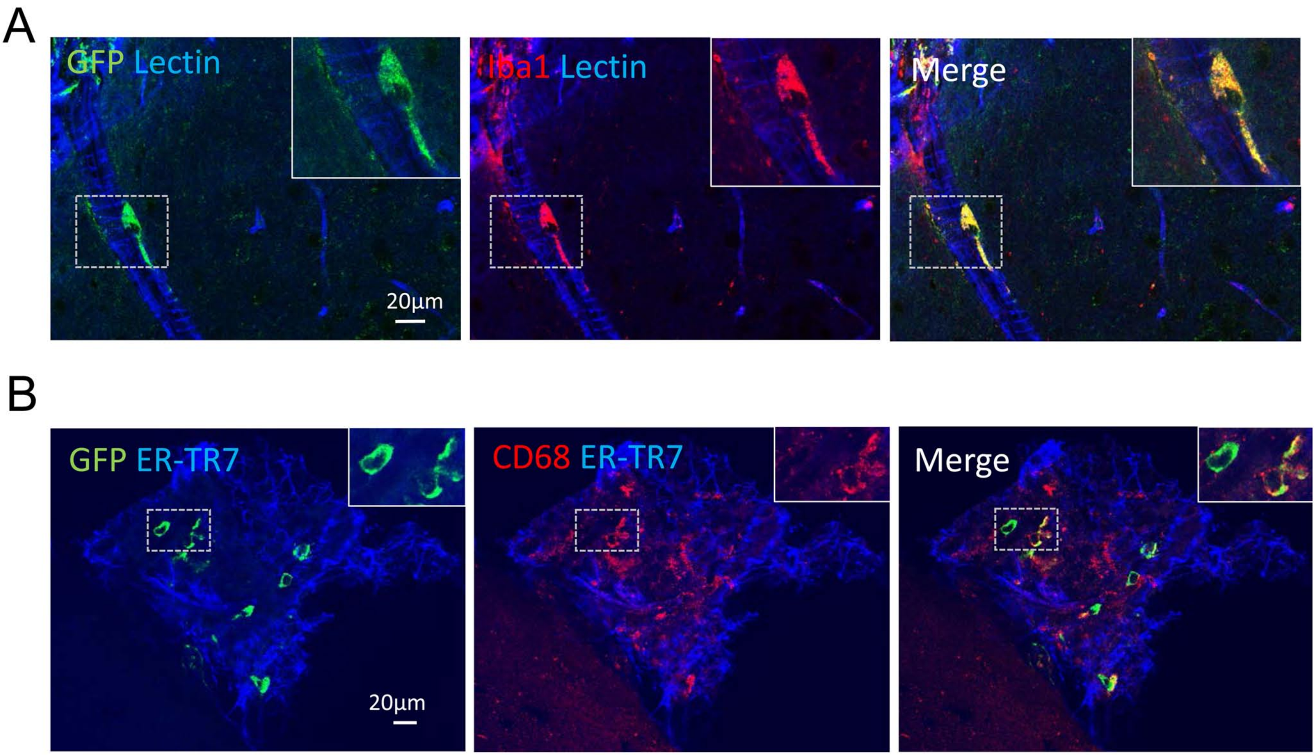

Supplementary Figure S3: GFP+ cells co-localize with macrophage markers in the perivascular space and meninges. A. Co-localization of GFP+ and Iba1+ cell within the perivascular space delineated by immunostaining for lectin. **B.** Co-localization of GFP+ and ER-TR7 (fibroblast-specific antigen) in the meninges.

Figure S4

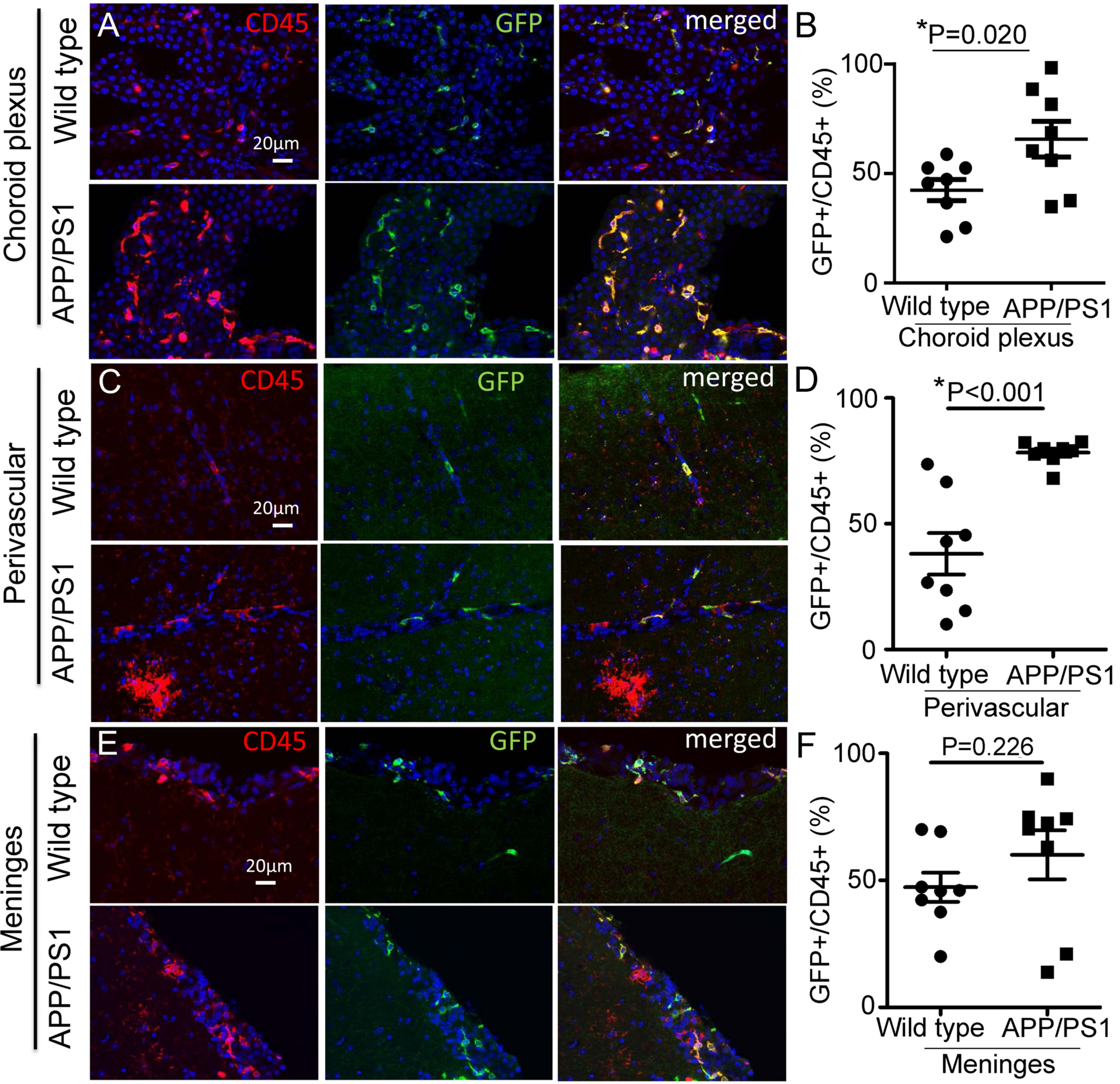

Supplementary Figure S4: Fate mapping strategy demonstrates increased contribution of definitive hematopoiesis-derived cells to the choroid plexus and perivascular space leukocyte population. Representative images demonstrating GFP expression in CD45+ cells in the choroid plexus in WTmTmG;Flt3-Cre and APP/PS1mTmG;Flt3-Cre mice between 10-17 months of age in the choroid plexus (A), perivascular space (D) and meninges (E); with quantitation of GFP expression in this cell population in the respective populations (B, D, and F). N=7-8 mice/group. P values depicted are by two-tailed t-test. * denotes P<0.05.

Figure S5

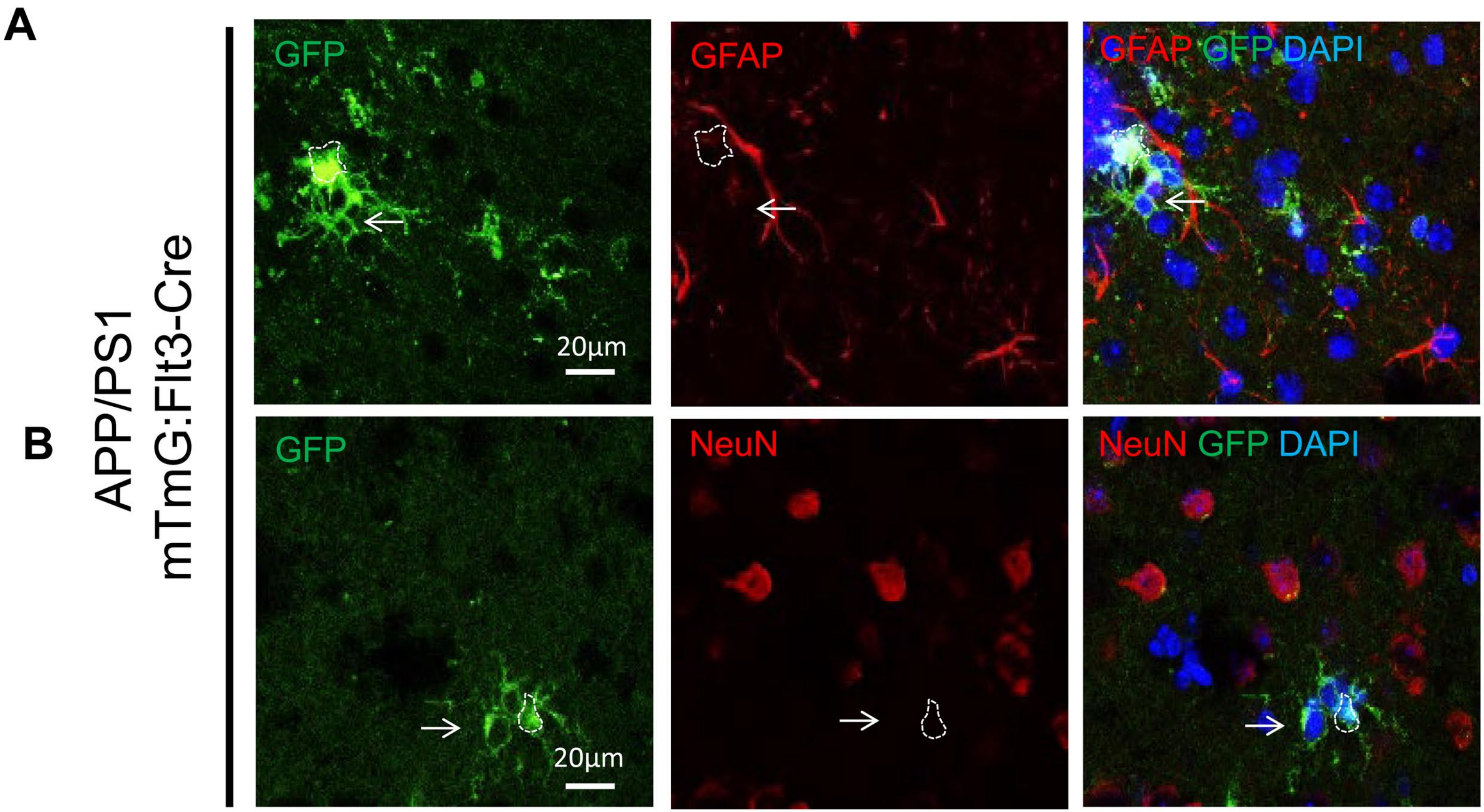

Supplementary Figure S5: GFP+ cells do not express astrocytic or neuronal markers. A, B. Cortical sections from APP/PS1 brain demonstrating lack of co-localization of GFP (arrows) with GFAP (astrocyte marker, A), and NeuN (neuronal marker, B).

Figure S6

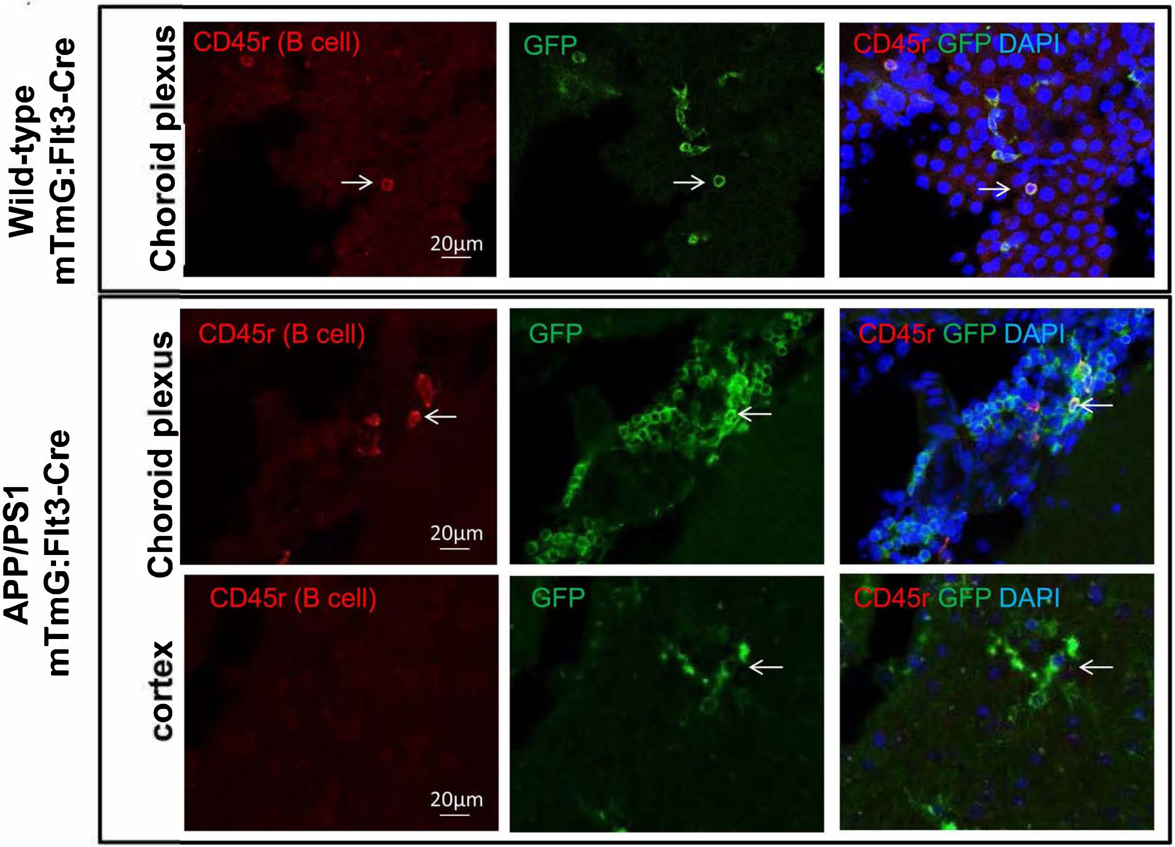

Supplementary Figure S6: GFP expressing B cells are detected in the choroid plexus but not in the brain parenchyma. Expression of CD45r (a B-cell expressed antigen) was detected in a fraction of GFP+ cells in the choroid plexus of both wild-type and APP/PS1 mice carrying the mTmG:Flt3Cre cassette, but not in the plaque-associated GFP+ cells in APP/PS1 mice.

Figure S7

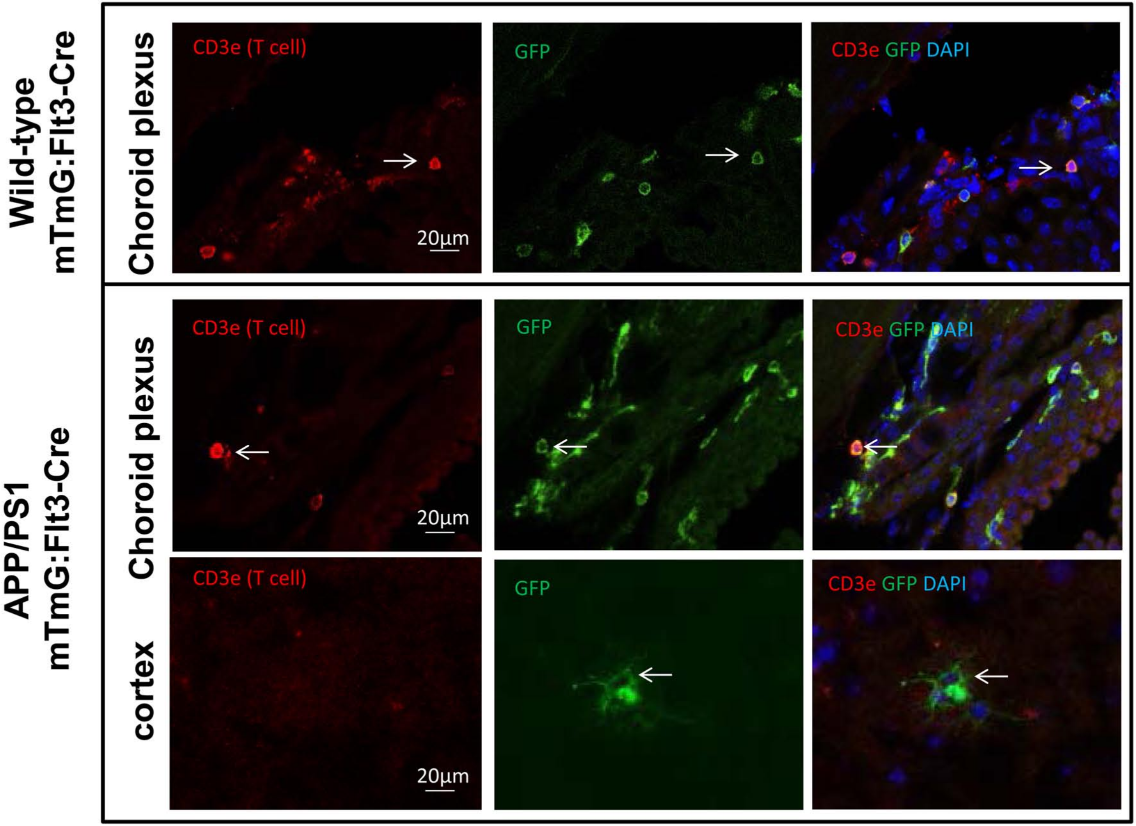

Supplementary Figure S7 : GFP expressing T cells are detected in the choroid plexus but not in the brain parenchyma. Expression of CD3e (a T-cell expressed antigen) was detected in a fraction of GFP+ cells in the choroid plexus of both wild-type and APP/PS1 mice carrying the mTmG:Flt3Cre cassette, but not in the plaque-associated GFP+ cells in APP/PS1 mice.

Supplementary Figure S8

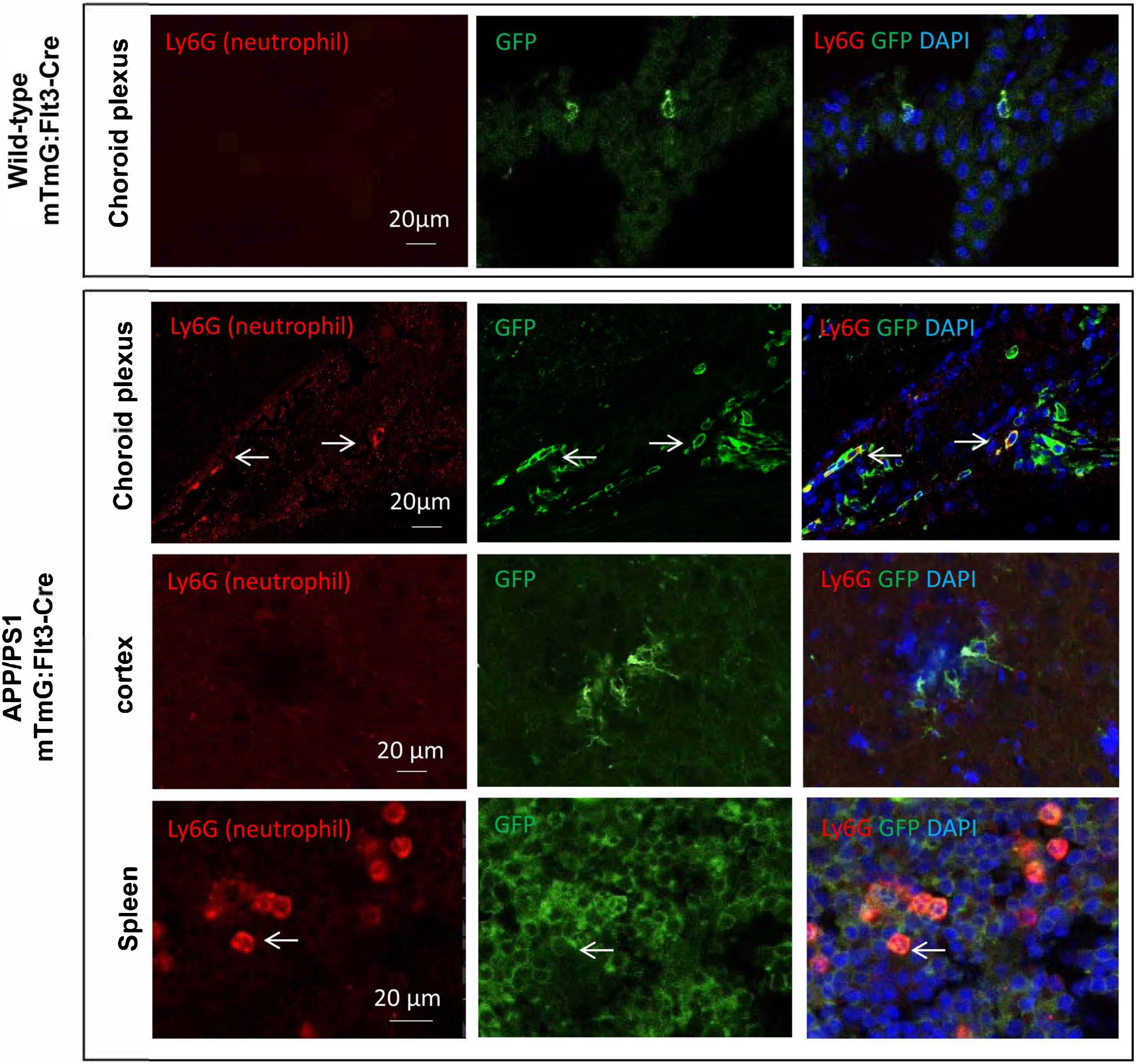

Supplementary Figure S8: Neutrophils are not detected in the brain parenchyma of mice. Expression of Ly6G (a neutrophil-expressed antigen) was detected in the choroid plexus from APP/PS1 mice carrying the mTmG:Flt3Cre cassette (2nd panel from top, arrow) but not in the brain parenchyma (3rd panel from top) or in wild-type (top panel). A section from spleen (bottom panel, arrow) is shown as positive control. N=6 mice were examined in each group.

Supplementary Figure S9

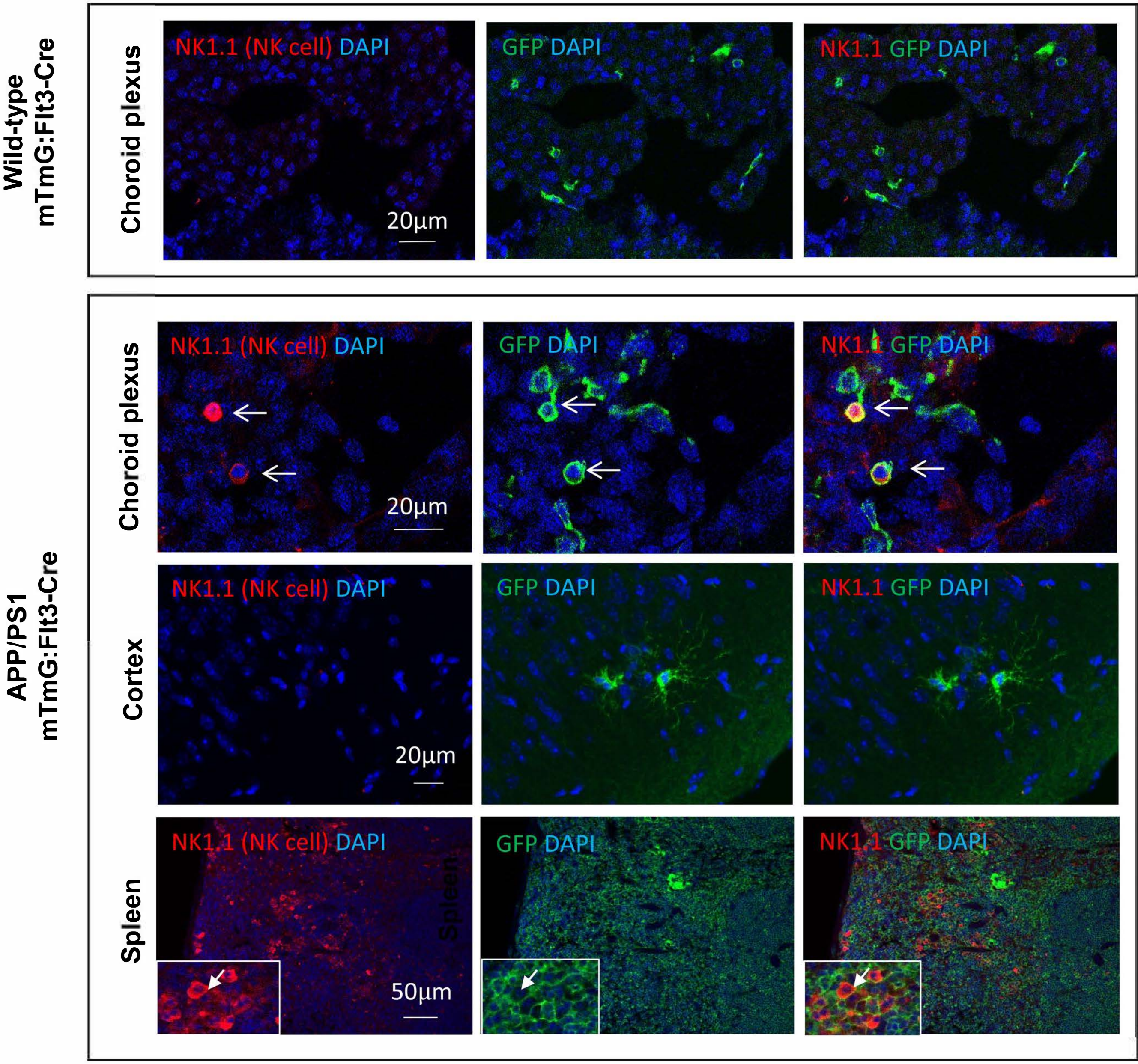

Supplementary Figure S9: NK cells are not detected in the brain parenchyma of mice. Expression of NK1.1 (a NK cell-expressed antigen) was detected in the choroid plexuses of APP/PS1 mice carrying the mTmG:Flt3Cre cassette (2nd panel from top, arrow) but not in the brain parenchyma (3rd panel from top) or in choroid plexus from wild type mice (top panel). A section from spleen (bottom panel, arrow) is shown as positive control. N=6 mice were examined in each group.

Figure S10

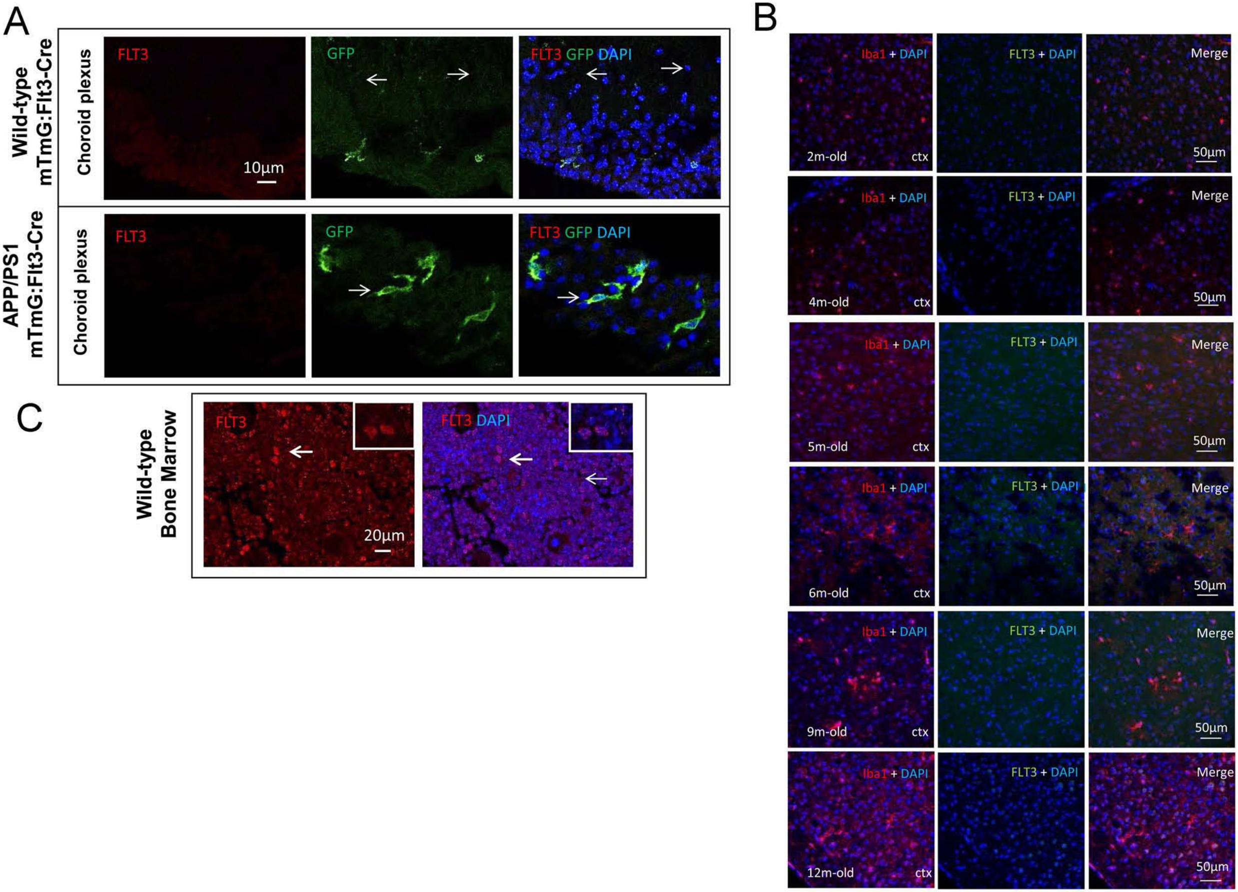

Supplementary Figure S10. FLT3 expressing cells are not detected in the brains of mice. A) Expression of FLT3 protein was not detected either aged (10 month old) wild-type (top panel, arrow) and APP/PS1 mice carrying the mTmG:Flt3Cre cassette (2nd panel from top, arrow). N=6 mice were examined in each group. **B)** Flt3 protein expression was not detected by immunohistochemistry in wild type brains at the indicated ages. **C)** A section from the bone marrow (arrow) is shown as positive control.

Figure S11

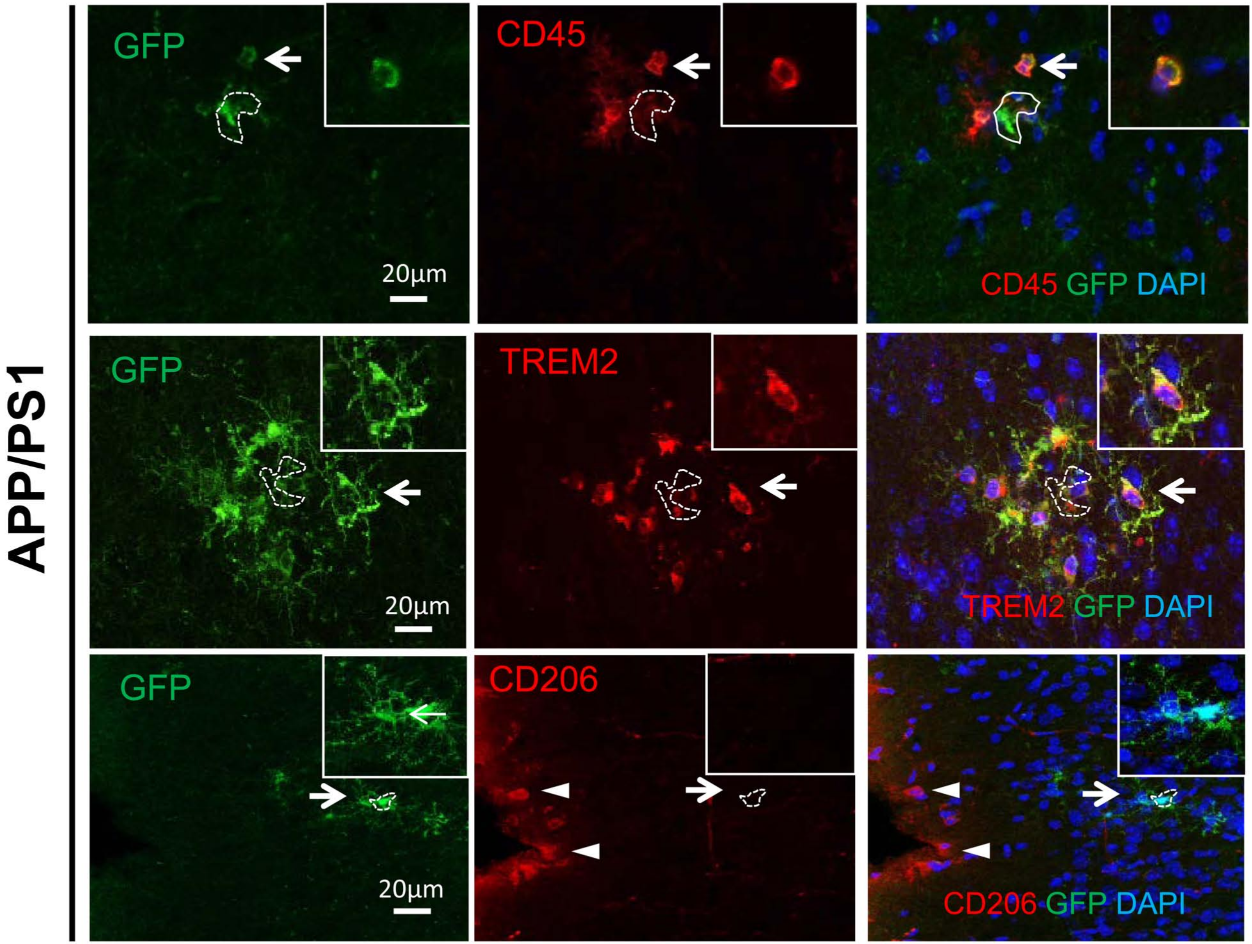

Supplementary Figure S11: GFP-tagged peripheral myeloid population-derived cells express macrophage and microglial markers. Representative cortical sections from aged APP/PS1mTmG;Flt3-Cre mice demonstrating that GFP+ cells seen adjacent to X-34 stained amyloid plaques (outlined) in the APP/PS1 transgenic mice express CD45 (top panel), TREM2 (middle panel) but not CD206 (bottom panel); see inset for cells pointed to with arrows. Arrowheads in D indicate CD206+ cells in the meninges.

Figure S12

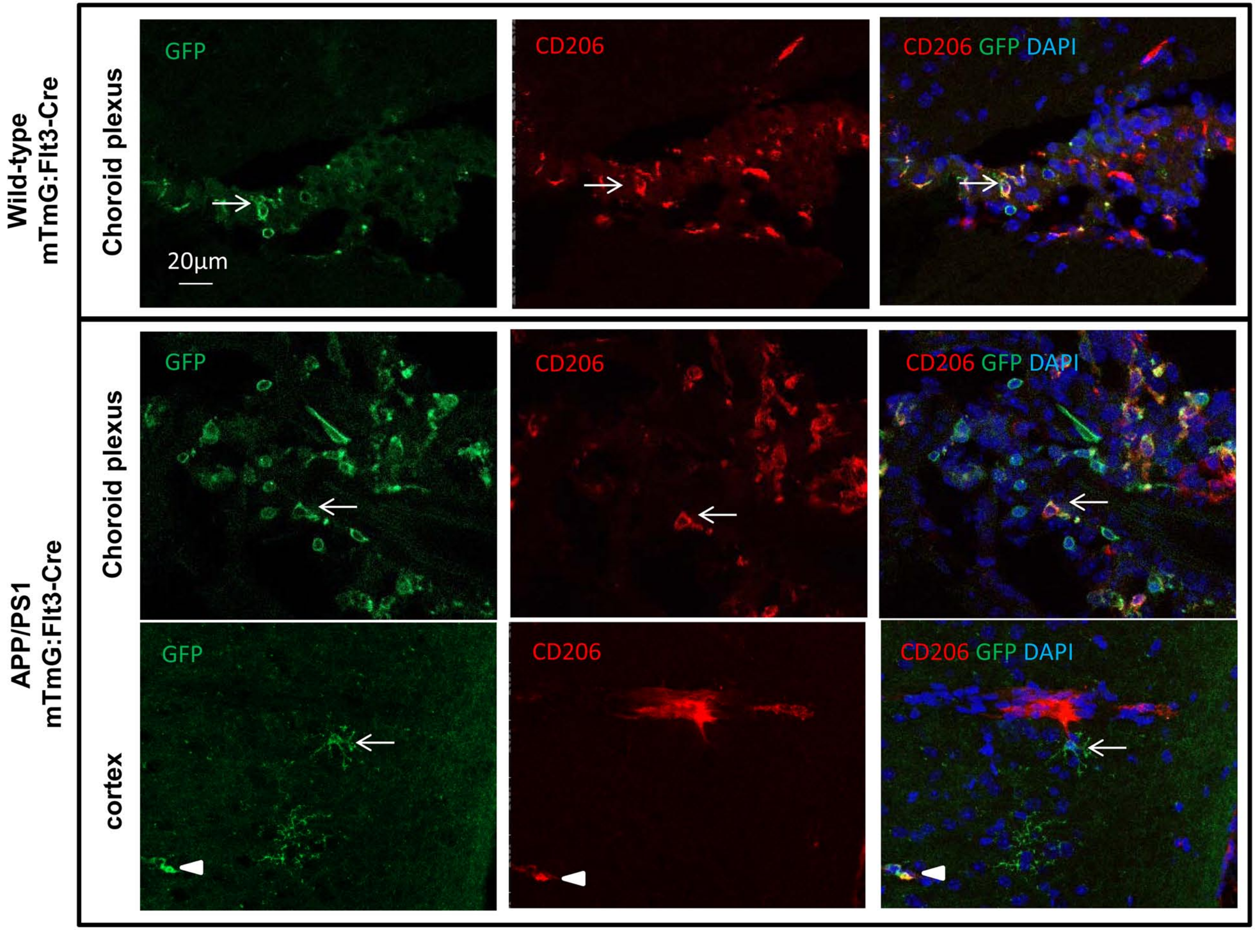

Supplementary Figure S12: GFP-positive CD206 expressing cells are detected in the choroid plexus and perivascular space, but not in the brain parenchyma. Expression of CD206 was detected in a fraction of GFP+cells in the choroid plexus of both wild-type and APP/PS1 mice carrying the mTmG:Flt3Cre cassette (see arrows, top and middle panel) and in the perivascular space (see arrowhead for GFP+ cell in the perivascular space, bottom panel) but not in the plaque-associated GFP+ cells in APP/PS1 mice (see arrow, bottom panel). N=6 mice were examined in each group.

Figure S13

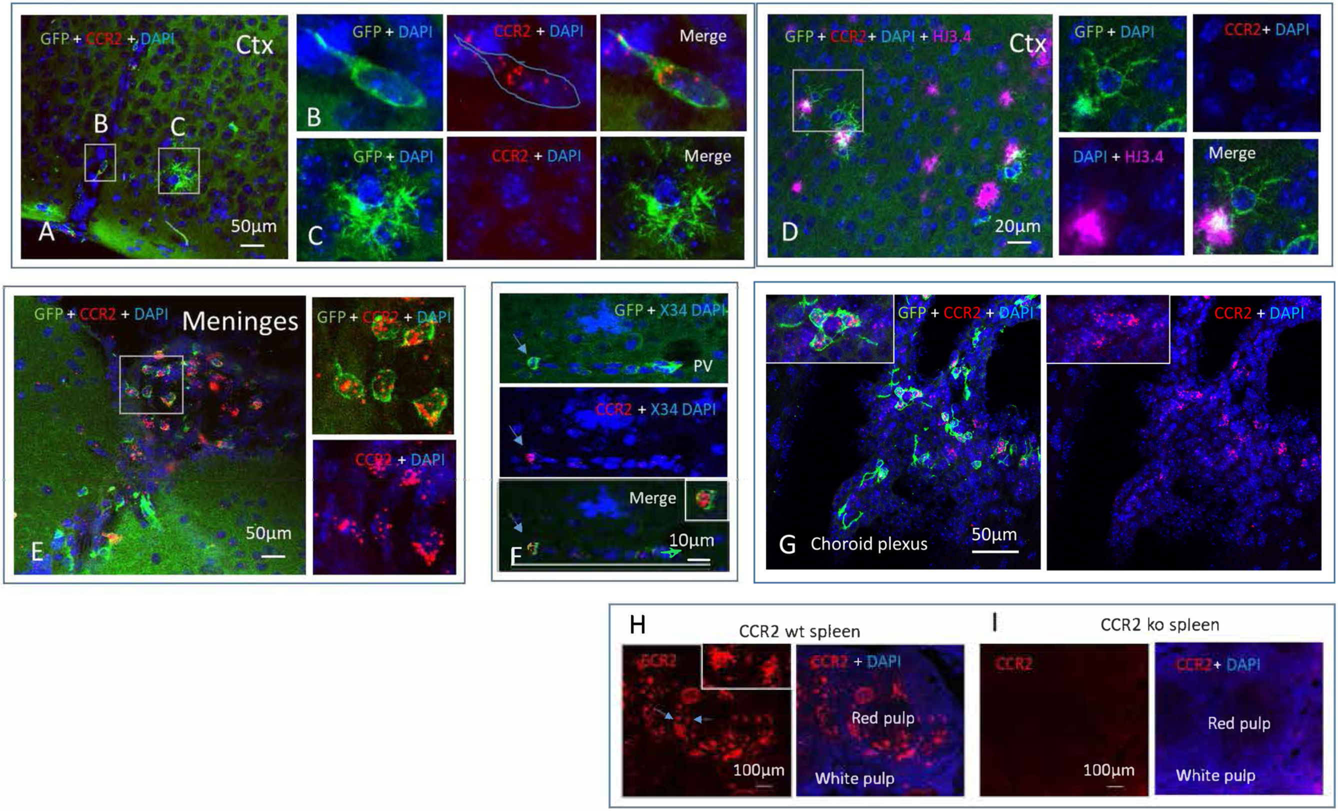

Supplementary Figure S13: GFP+ cells express Ccr2 in the perivascular space, meninges and choroid plexus, but not in the brain parenchyma. A-I. In situ hybridization was performed to detect *Ccr2* transcript expression in APP/PS1 brains harboring the fate-mapping cassette. *Ccr2* expression (red) is observed in GFP+ perivascular macrophages (seen in A, B, F), meningeal macrophages (E), and choroid plexus macrophages (G), but not in GFP+ peri-plaque macrophages (C, D). Spleen tissue from a *Ccr2* null mouse (I) and wild type (H) is shown as a control.

Supplementary Figure S14

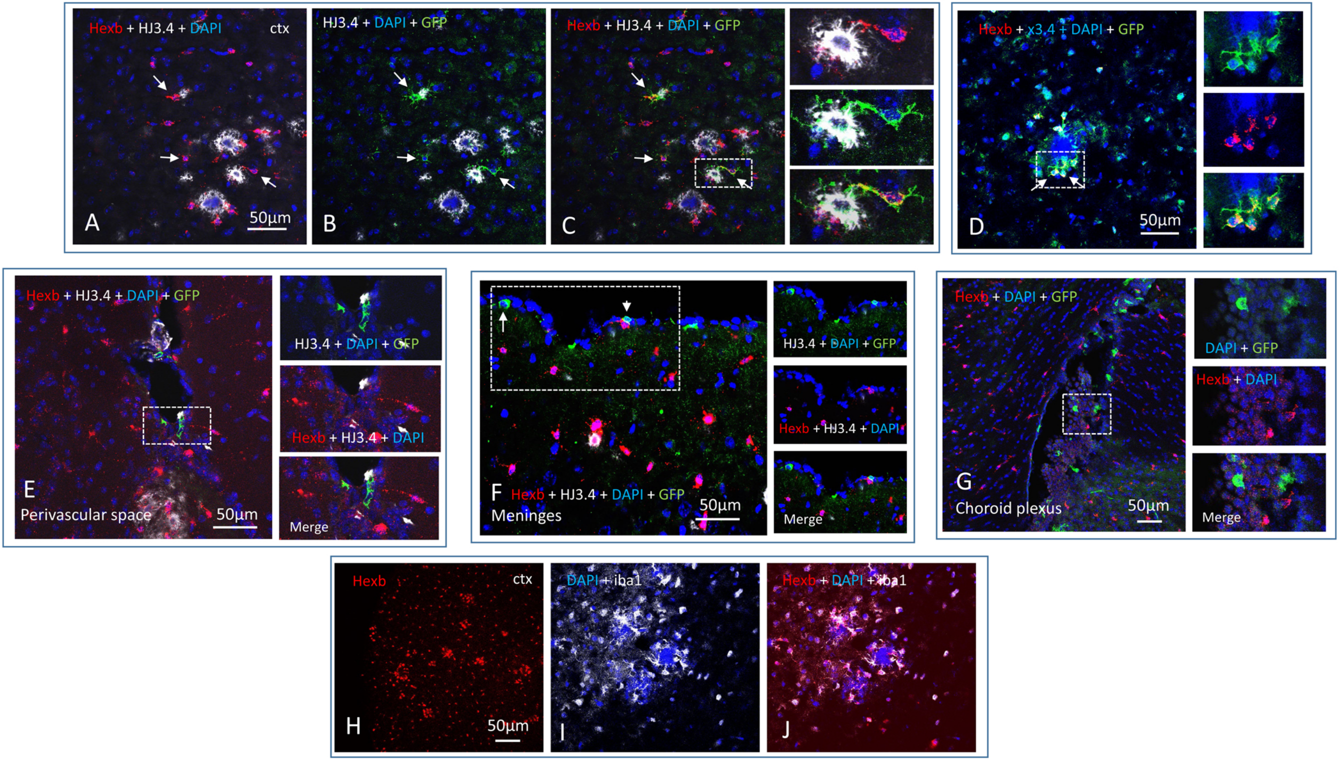

Supplementary Figure S14: GFP+ cells express Hexb in the brain parenchyma, but not in the perivascular space, meninges and choroid plexus. A-J. In situ hybridization was performed to detect *Hexb* transcript expression in APP/PS1 brains harboring the fate-mapping cassette. *Hexb* expression is observed in endogenous microglia and GFP+ peripheral monocyte-derived peri-plaque macrophages (A-D) but not observed in GFP+ perivascular macrophages (seen in E), meningeal macrophages (F), and choroid plexus macrophages (G). Hexb expressing cells also express iba1+ (H, I, L). Dotted lines indicate areas shown in magnified images in A-G.

Supplementary Figure S15

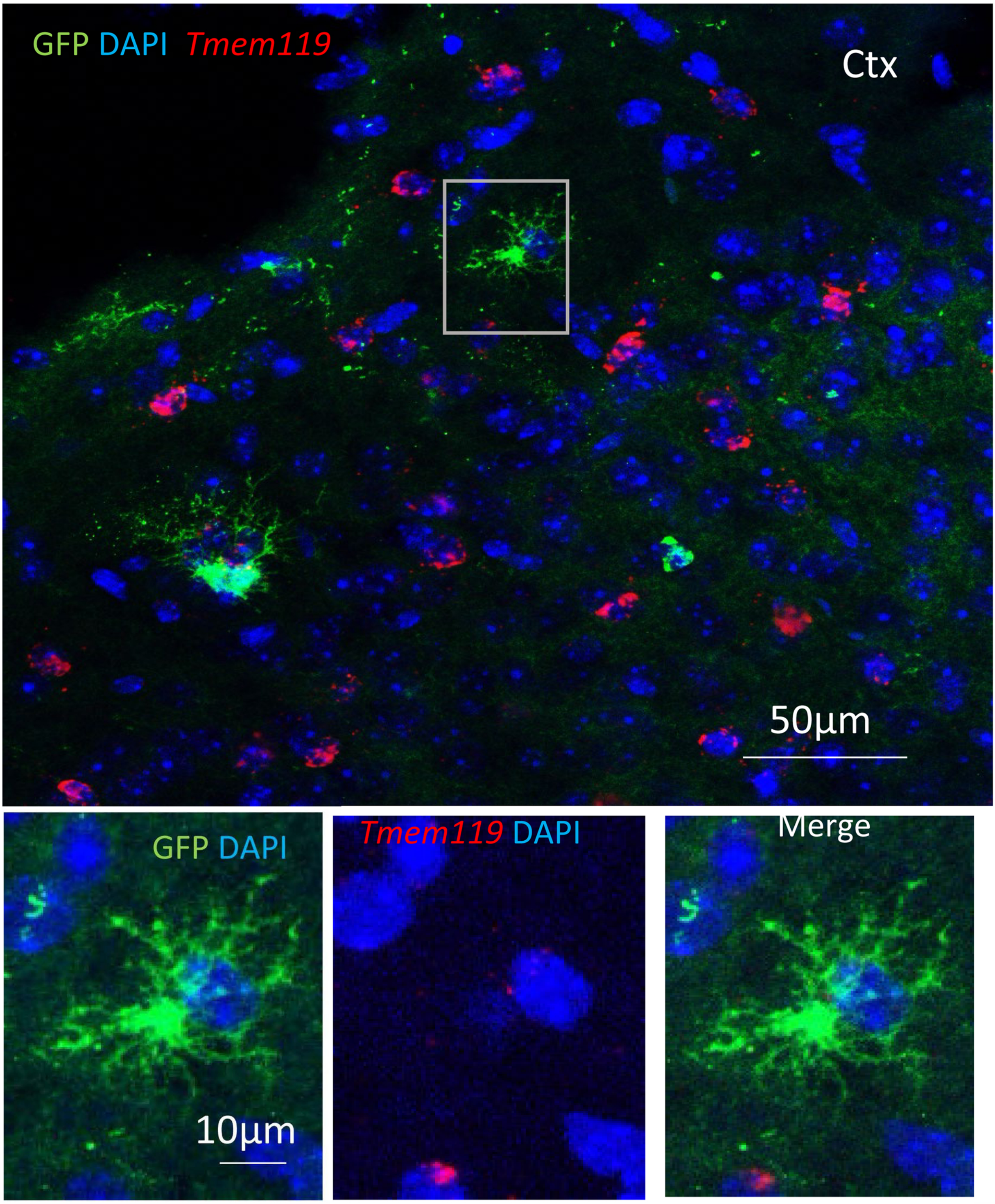

Supplementary Figure S15: GFP+ cells do not express TMEM119, a microglia-specific transcript. In situ hybridization was performed to detect *Tmem119* transcript expression in APP/PS1 brains harboring the fate-mapping cassette. TMEM119 expression is observed in endogenous microglia but not in GFP+ peri-plaque macrophages.
